# SurvdigitizeR: an algorithm for automated survival curve digitization

**DOI:** 10.1186/s12874-024-02273-8

**Published:** 2024-07-13

**Authors:** Jasper Zhongyuan Zhang, Juan David Rios, Tilemanchos Pechlivanoglou, Alan Yang, Qiyue Zhang, Dimitrios Deris, Ian Cromwell, Petros Pechlivanoglou

**Affiliations:** 1 Child Health Evaluative Sciences, Peter Gilgan Centre for Research and Learning, The Hospital for Sick Children, Toronto, ON Canada; 2 Biostatistics Division, Dalla Lana School of Public Health, University of Toronto, Toronto, ON Canada; 3 Lassonde School of Engineering, York University, Toronto, ON Canada; 4 Michael G. DeGroote School of Medicine, McMaster University, Hamilton, ON Canada; 5 Canada’s Drug Agency, Ottawa, ON Canada; 6 Institute of Health Policy, Management and Evaluation, Dalla Lana School of Public Health, University of Toronto, Toronto, ON Canada

**Keywords:** Automated Digitization, Meta-analysis, Survival Analysis, Kaplan–Meier Curve, R Package, Shiny application

## Abstract

**Background:**

Decision analytic models and meta-analyses often rely on survival probabilities that are digitized from published Kaplan–Meier (KM) curves. However, manually extracting these probabilities from KM curves is time-consuming, expensive, and error-prone. We developed an efficient and accurate algorithm that automates extraction of survival probabilities from KM curves.

**Methods:**

The automated digitization algorithm processes images from a JPG or PNG format, converts them in their hue, saturation, and lightness scale and uses optical character recognition to detect axis location and labels. It also uses a k-medoids clustering algorithm to separate multiple overlapping curves on the same figure. To validate performance, we generated survival plots form random time-to-event data from a sample size of 25, 50, 150, and 250, 1000 individuals split into 1,2, or 3 treatment arms. We assumed an exponential distribution and applied random censoring. We compared automated digitization and manual digitization performed by well-trained researchers. We calculated the root mean squared error (RMSE) at 100-time points for both methods. The algorithm’s performance was also evaluated by Bland–Altman analysis for the agreement between automated and manual digitization on a real-world set of published KM curves.

**Results:**

The automated digitizer accurately identified survival probabilities over time in the simulated KM curves. The average RMSE for automated digitization was 0.012, while manual digitization had an average RMSE of 0.014. Its performance was negatively correlated with the number of curves in a figure and the presence of censoring markers. In real-world scenarios, automated digitization and manual digitization showed very close agreement.

**Conclusions:**

The algorithm streamlines the digitization process and requires minimal user input. It effectively digitized KM curves in simulated and real-world scenarios, demonstrating accuracy comparable to conventional manual digitization. The algorithm has been developed as an open-source R package and as a Shiny application and is available on GitHub: https://github.com/Pechli-Lab/SurvdigitizeR and https://pechlilab.shinyapps.io/SurvdigitizeR/.

**Supplementary Information:**

The online version contains supplementary material available at 10.1186/s12874-024-02273-8.

## Background

Health technology assessment is a tool used for evaluating the clinical and economic impact of healthcare interventions. It heavily relies on statistical methods such as decision analytic modeling and (network) meta-analysis, which utilize time-to-event data [1, 2]. Also known as survival data, time-to-event data tracks the time until a certain event of interest, such as disease progression or death. Such types of data usually inform estimate of relative effectiveness and cost-effectiveness of various medical treatments and interventions [3]. In economic evaluations, time-to-event data are frequently used to estimate key model parameters, typically through the application of parametric survival analysis [4].

Gaining access to individual patient-level data (IPD) on survival outcomes, especially from randomized controlled trials can be challenging due to concerns about confidentiality and proprietary rights. Often, researchers only have access to aggregate data summarized in tables and figures from publications and reports. A Kaplan–Meier (KM) curve, a fundamental tool in medical research, plots the probability of an event of interest (typically time-to-event data, such as survival) over time, allowing for the visualization of the estimated survival function. To address the issue of limited access to IPD, methods have been developed in recent years to reconstruct IPD from these published materials, including KM curves [5, 6]. This reconstruction process combines digitization and heuristic algorithms and involves several steps.

1. Manually digitizing a KM survival curve to extract the time points where probability changes and the magnitude of those changes.
2. Extracting the number of people at risk per unit of time from the original publication or report. Such tables are commonly reported alongside KM curves.
3. Utilizing a heuristic algorithm such as the one developed by Guyot et al. [5] to construct synthetic IPD data that match the digitized data and the “at risk” table of people at risk.
4. Applying standard survival analysis methods to the synthetic data to inform the decision models or network meta-analysis.

Recently developed methods, such as IPDfromKM, provide a two-step workflow for IPD reconstruction, where the initial step requires manual data point extraction and then perform downstream analysis based on digitized data points [6]. Manual digitization of data using proprietary or open-source software like Digitize-It or Engauge Digitizer [7, 8], comes with several limitations. First, the process requires technical training, which can be time-consuming. For large meta-analyses with many plots, the amount of human labor needed and hence the costs can be significant. Moreover, the reliability and reproducibility of the analysis results is potentially compromised because of variability between different individuals performing the extraction. Differences in interpretation and data extraction techniques can lead to inconsistent results. Also, access to some proprietary software may be limited, posing difficulties for peers wishing to reproduce research findings. These issues highlight the need for more efficient and standardized methods of IPD reconstruction and analysis.

The primary objective of this study is to develop an open-source computer algorithm that automates the digitization of KM curves from image files, minimizing user input while accurately extracting time points and survival probabilities. A secondary objective is to evaluate the accuracy, reliability, efficiency and validity of the algorithm through a simulation study and using real world published survival curves, where the automated digitization output is compared to that obtained through manual digitization. Finally, we facilitate the use and accessibility of the algorithm by providing an R package and a Shiny application.

## Implementation

### The automated digitization algorithm

**Fig. 1:**
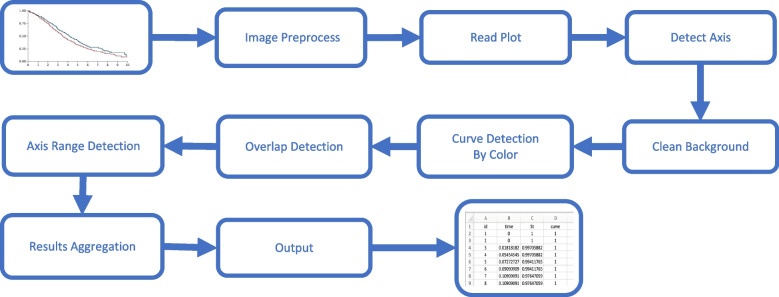
Algorithmic process for automated digitization

The algorithm begins by reading an image file (Step 1) and identifying the location of the plot axes (Step 2). Subsequently, the algorithm isolates and removes any background pixels and text from the plot (Step 3), and clusters the pixels into curves based on colour (Step 4). Following this, it addresses missing or overlapped pixels (Step 5), isolates the curve information (Step 6), and maps the x and y pixel locations to the actual time and survival values, respectively (Step 7). Figure 1 illustrates the algorithmic process for automated digitization, and further details on each step of the algorithm is provided in the methods section. Below we present the algorithm steps and the associated R functions from the SurvdigitizeR package.

1. *Loading KM curve(s):* The JPG or PNG file containing the KM survival curve(s) is loaded. This results in a Hue, Saturation, Lightness (HSL) scale of the image for further processing.
2. *Identifying axes location:* The algorithm determines the location of the plot’s x and y axes. The image is then cropped based on axes locations to keep only the relevant plot region.
3. *Removing non-KM portions of the plot:* This step removes non-essential elements from the plot such as background pixels and text. Lightness thresholds and optional Optical Character Recognition (OCR) are used to separate the KM curves.
4. *Detecting curves:* This step identifies and separates the different survival curves in the image based on color. The number of curves to be detected is provided as a parameter in the main function.
5. *Addressing potential overlap:* If multiple curves overlap, the algorithm rectifies missing or overlapping pixels. This ensures the completeness of curve information detected due to overlaps.
6. *Isolating curves:* For KM plots with more than one curve, the algorithm isolates unique probability (y-axis) values for each curve, preparing them for mapping to survival probability values.
7. *Mapping pixels to actual scales:* The algorithm ascertains how x and y pixels correspond to time and survival values. This function leverages the scale on the plot’s axes to translate pixel values to actual values: survival time and probabilities.
8. *Merging x and y-axis values with curves:* Lastly, this step merges the x and y-axis pixel values with the recognized curves from previous steps to generate the final data frame, including time points, survival probabilities, and curve identities. The output of SurvdigitizeR is a structured data frame that carries the digitized information.

### SurvdigitizeR package and Shiny application

We combined the functions above to an R package named SurvdigtizeR For the development of the package we used R 4.2.2. The survival_digitize() function is a comprehensive main function for digitizing KM curves from image files, which is a wrapper for the end-to-end digitization process, effectively managing the entire flow from image loading to the final output. It accommodates various parameters to customize the digitization procedure, such as background lightness threshold, OCR settings, the number of curves in the image, axis label information, and more. The R Package is available on GitHub: https://github.com/Pechli-Lab/SurvdigitizeR.

We developed a Shiny application to enhance accessibility and ease of use of the SurvdigitizeR package, This application offers an intuitive web-based interface for digitizing KM curves from image files and does not require any software installation. The Shiny application is accessible at: https://pechlilab.shinyapps.io/SurvdigitizeR/. A user guide can be found on the Shiny App website and in the supplementary material Shiny Application section. The supplementary Fig. S1 illustrates the user interface.

### Simulation studies

To assess the performance of the digitizer algorithm, we conducted a study using KM curves generated from simulated data. We compared the algorithm’s output to the known simulated probabilities underlying each KM plot image. Utilizing simulated data also enabled us to validate the algorithm’s performance under various conditions, such as applying different graphical engines to generate the KM plots, generate KM curves using data with various sample sizes, modifying the number of curves per image, and the presence of censoring indicators in the KM curves.

We generated survival time datasets for 1000 individuals, with event times randomly drawn from an exponential distribution at a rate of 0.01. In order to assess the effect of the presence of censoring markers on the KM curves on the performance of automatic digitization, we introduced scenarios with right censoring, which happens when the study follow-up duration is shorter than the event time. Censoring times, uncorrelated with survival times, were drawn from an exponential distribution with a rate of 0.005. If an individual’s censoring time is less than their event time, we record the censoring time and change the individual’s event status to 0: censored, indicating that the event did not occur during the observed period.

For plot images with two or more KM curves, we randomly assigned individuals into groups. The random assignment was a conservative assumption that resulted in considerable overlap between KM curves in a plot. When generating KM plots, we varied the number of individuals (*N* = 25, 50, 150,250, 1000), the number of KM curves in a plot (1, 2, 3), and whether individuals can be censored. Also, We compared two KM curve plotting engines, R base vs ggplot [9, 10]. In total, we generated 60 plots with a total of 120 KM curves. All images were saved as JPEG files.

We prespecified algorithm inputs that were unique to each plot image, such as the number of curves in an image or the size of the x and y-axis increments. In addition, there are algorithm inputs that were common for all images in the simulation study, such as the sensitivity in removing background distortion, were set to existing defaults that we found in the development of the digitizer algorithm to produce good digitizing results.

We evaluated the algorithm’s digitization accuracy at 100 time checkpoints for each curve, spaced evenly from 0—the common starting point of study time on the x-axis—to the maximum study time value of a specific curve. The output of the algorithm includes digitized survival probabilities. For each of the 100 time checkpoints, there is a corresponding digitized survival probability and a true survival probability. Since the time checkpoints may not be exactly the digitized time point, we assign the best candidate probabilities to the time check points using following probability remapping approach. We assumed that survival probabilities decrease monotonically, which implies that the KM curve can only extend rightward (time increases) and downward (probability decreases on the plot). We used digitized data in our analysis: v_time: timepints of the original digitized survival data, v_surv: correspoinding survival probabilities at each time point in v_time, v_time_check: time points for which we need to determine the survival probabilities. For each time point t in v_time_check, we determined the corresponding survival probability S(t) as follows:

1. Identify the subset of time points in v_time that are less than or equal to t, denoted as $T \le t$ 
2. If all time points in v_time are less than or equal to t, then set S(t) = 0, indicating no survivors beyond time t.
3. Otherwise, find the lasts time point within $T \le t$, denoted as t_max. Use the corresponding survival probability$S\left({t}_{max}\right)$ in v_surv.

  

After the probabilities remapping, we calculated the root mean squared error (RMSE) for each curve:

$$RMSE=\sqrt{\left\{mean\left({\left(digtizer est. survival probability-true survival probability\right)}^{2}\right)\right\}}$$

For a plot containing more than 1 curve, we reported the averaged RMSE of 2 or 3 curves on the plot. We provided summaries of the RMSE stratified by sample size, the number of images in a plot, the censoring marker, and the R package used to generate each plot. We also generated plots that overlayed the digitized survival probability over the survival probability estimated directly from the data used to generate the KM plot.

To validate the algorithm’s performance against the current standard practice of manual digitization, we compared the automated digitization algorithm’s output with the results of manual digitization of the simulated curves. The KM survival curves generated in the simulation study were manually digitized by one extractor after receiving training on using the Engauge Digitizer [8]. For the 60 simulated plots, we calculated the RMSE between the manual digitization results and the true survival probability. Then, we compared these results with the automated digitization outcomes in the same context. Given the paired nature of our data, where each plot was digitized both manually and automatically, a paired t-test was conducted to compare the two sets of RMSE values.

### Real world validation

To assess the performance of our automated digitization algorithm in a real-world context, we conducted tests on a collection of eight published Kaplan–Meier (KM) plots in order to measure its agreement with manual digitization. We selected plots which contained two curves in each plot. These plots, which were generated using a variety of tools and software, exhibited more intricate features than the simulated curves addressed in the preceding section, presenting specific challenges for axis and KM curve detection, such as non-standard axis colors, censoring markers on the KM curves, and unrelated text overlapping the graphs.

In contrast to simulation studies, retrieving true survival probabilities from these real-world plots was infeasible. Instead, KM curves were manually digitized by one trained extractor using Enguage Digitizer [8]. To quantify the agreement between the manual digitizations and those produced by our algorithm, we employed Bland–Altman plots [11]. These plots are instrumental in visualizing the differences between two measurement methods against their average value and are particularly useful for evaluating consistency across various instances.

For each KM curve, we computed the differences in survival probabilities between the automated digitization and the manual approach, along with their mean and the standard deviation of these differences at 100 time checkpoints. Similar to the approach described in RMSE calculation, the time points were constructed by evenly spacing intervals from 0 to the maximum study time. With this data, we set the Bland–Altman plot limits to ± 1.96 standard deviations from the mean difference, delineating the range where we would expect 95% of the differences to fall if the two methods agreed. Bland–Altman plots were then generated for the KM curves in each test case, displaying the mean difference as a solid line and the agreement limits as dashed lines. This procedure was consistently applied to eight test plot cases which contain a total of 16 KM curves to validate the algorithm’s performance in real-world conditions.

We further strengthened our validation process by incorporating Kendall’s Tau correlation [12]. This statistical method was applied to the digitization of each Kaplan–Meier curve to quantify the concordance between the automated and manual digitization methods. Specifically, within each test plot, there are two curves. For every curve, we compared the two digitization methods (manual vs. automated digitization) of Kaplan–Meier curves. We calculated Kendall’s Tau correlation coefficient, which measures the strength and direction of the association between the two sets of rankings derived from the digitized survival probabilities at various time points. To create equal-length, paired sets of digitized probabilities, we used the time points from manual digitization as a reference and extracted the corresponding survival probabilities from the automated digitization’s output. Kendall’s Tau provides a value ranging from -1 to 1, where values closer to 1 indicate a strong positive correlation, suggesting high agreement between the manual and automated digitizations. Conversely, values closer to -1 would indicate a strong negative correlation, signifying disagreement. A value of 0 would imply no correlation.

## Results

### Simulation study results

Table 1 presents the RMSE for both manual and automated digitization methods under various conditions. Overall, automated digitization produced lower RMSE, achieving a lower average RMSE (0.012) compared to the manual process (RMSE = 0.014). The RMSE was lower for the automated digitization in 37 out of 60 simulated plots. We performed a one-sided paired t-test to test the hypothesis that automated digitization results in a lower RMSE than manual methods. The *p*-value obtained was 0.026, which is statistically significant under the conventional 0.05 threshold. This suggests a trend toward lower RMSE for automated versus manual digitization.

**Table 1:** Comparative analysis of average RMSE across various plot image categories: manual vs. automated digitization

Category	N Plots	RMSE		Automated Performing Better (%)
		**Manual**	**Automated**	
Overall	60	0.014	0.012	62
Censoring Marker: FALSE	30	0.015	0.009	80
Censoring Marker: TRUE	30	0.013	0.016	43
Sample Size: 25	12	0.017	0.013	83
Sample Size: 50	12	0.016	0.011	67
Sample Size: 150	12	0.013	0.012	50
Sample Size: 250	12	0.012	0.012	50
Sample Size: 1000	12	0.014	0.014	58
Number of Curve on Plot: 1	20	0.014	0.010	75
Number of Curve on Plot: 2	20	0.013	0.012	60
Number of Curve on Plot: 3	20	0.016	0.014	50
Package to Generate Plot: base	30	0.014	0.011	67
Package to Generate Plot: ggplot2	30	0.015	0.014	57

**Fig. 2:**
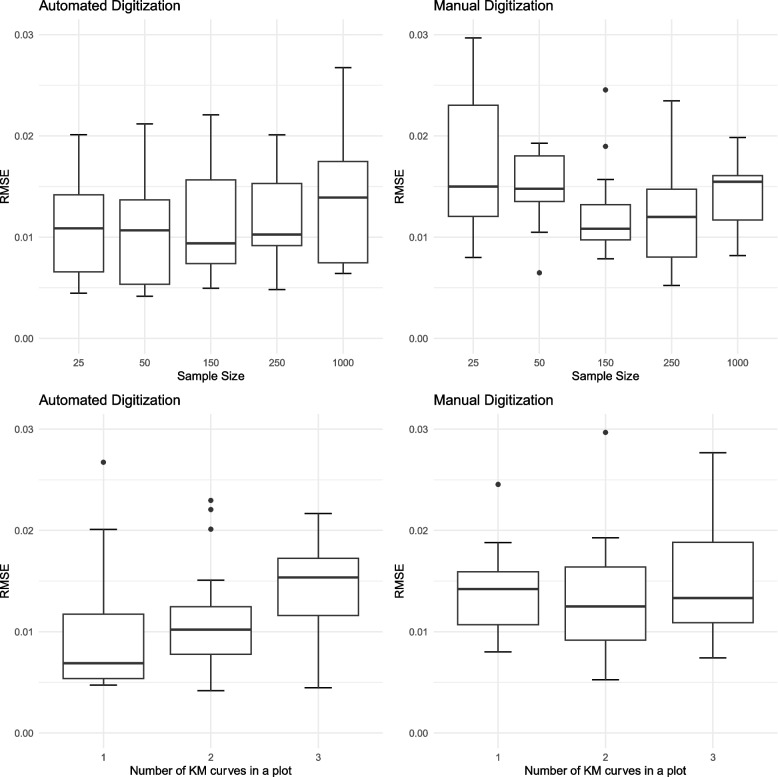
Boxplot for overall RMSE for automated digitization and manual digitization, according to on sample size and number of KM curves in the plot

In our comparative analysis, the role of sample size in influencing the performance of manual versus automated digitization was demonstrated. Table 1 reveals that for sample sizes of 25, 50 and 150, automated digitization outperformed manual methods, achieving lower average RMSE values of 0.013, 0.011 and 0.012, respectively, compared to 0.017, 0.016 and 0.013 in the manual process. This indicates that automated methods handle same to moderate-sized datasets more effectively. However, as the sample size increases, the differences in performance between the two methods become less pronounced. For example, at a sample size of 250 and 1000, both automated and manual digitization methods produced similar performance with same average RMSE values 0.012 and 0.014, an overall comparison of results can be referred in Fig. 2.

**Fig. 3:**
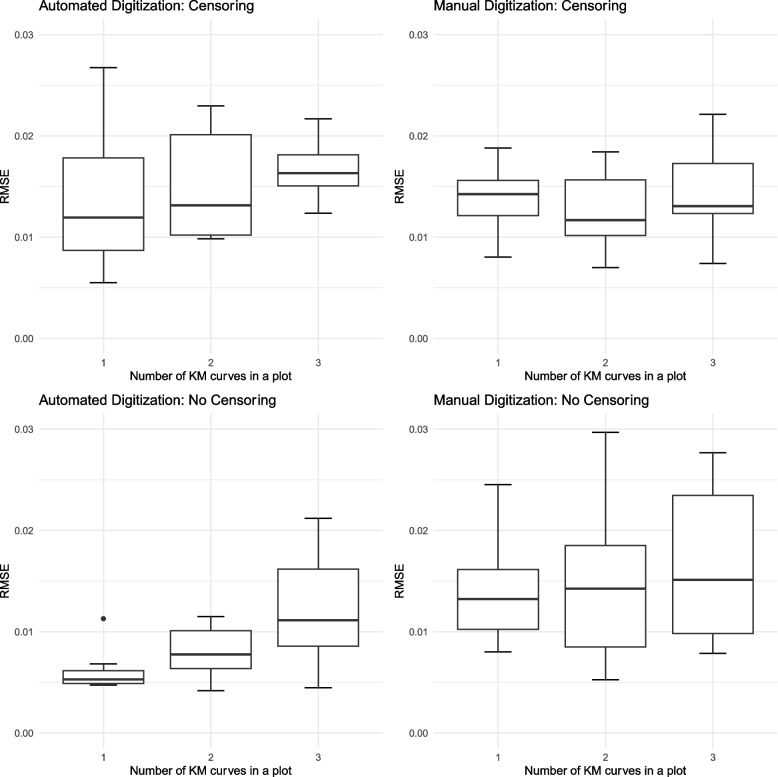
Boxplot for RMSE for automated digitization and manual digitization according to censoring marker existence

In curves where a censoring marker was not present, the automated digitization approach outperformed manual digitization, achieving a lower average RMSE (0.009 vs. 0.01, respectively) as shown in Table 1. Furthermore, Fig. 3 demonstrates that automated digitization performs well in the absence of censoring markers. However, when censoring markers were present, manual digitization slightly outperformed automated digitization (0.013 vs. 0.016, respectively), and the average RMSE across curves showed a larger variance, as illustrated in Fig. 3.

**Fig. 4:**
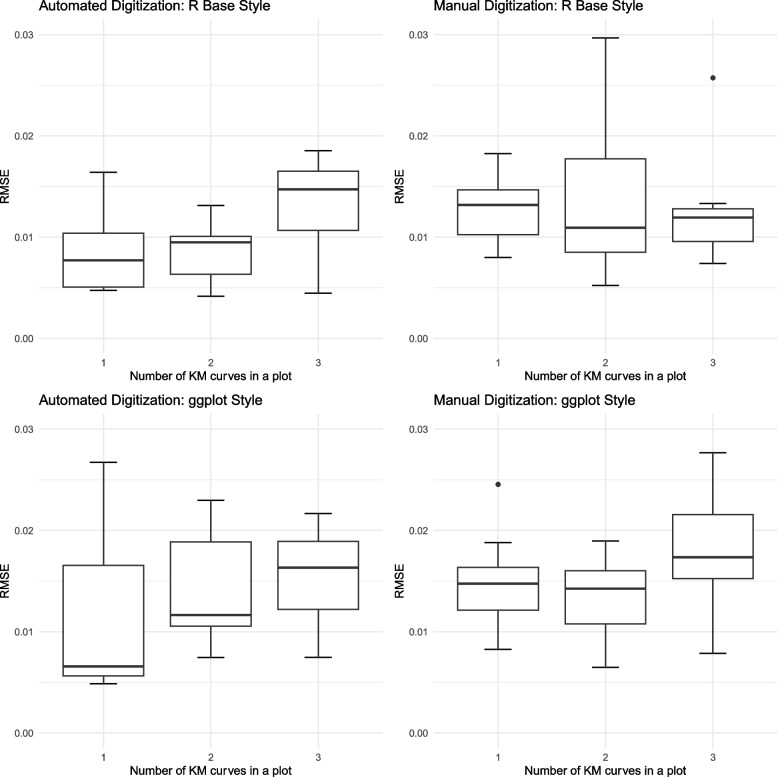
Boxplot for RMSE comparisons between auto digitization and manual digitization according to plot style: R base and ggplot2

Finally, in plots developed using the R base or ggplot engines, the automated process was found to be more accurate than manual digitization, achieving a lower average RMSE (0.011 vs. 0.014) and (0.014 vs 0.0015), respectively (Fig. 4). When the number of curves on a plot are 1 and 2, automated digitization can have decent performance compared with manual digitization. When there are 3 curves on a plot, manual digitization performs better and causes less deviation from the true values as reflected by a lower RMSE, indicating its superiority in more complex plotting scenarios.

### Real-world validation results

The results in Table 2 reveal a high level of agreement between manual and automated digitization of Kaplan–Meier curves, as measured by Kendall’s Tau. Across all cases and curve types—Overall Survival (OS) and Progression-Free Survival (PFS)—the Kendall Tau values are consistent, with all values equal to or exceeding 0.99. This strong positive correlation indicates that the automated digitization closely aligns with the manual approach, affirming the algorithm’s accuracy and reliability in replicating manual digitization. For a more detailed visual assessment of the agreement between manual and automated digitization methods, Bland–Altman plots for each case are provided in the supplementary Fig. S2-S9.

**Table 2:** Curve-specific agreement comparison between manual and automated digitization through Kendall Tau (KT)

Plot ID	Kendall Tau	Kendall Tau
	**Curve 1**	**Curve 2**
1	0.996	0.996
2	0.997	0.996
3	0.992	0.982
4	0.994	0.991
5	0.991	0.994
6	0.989	0.996
7	0.997	0.990
8	0.998	0.998

## Discussion

This study presented an automated digitization algorithm for survival probability curves and demonstrated its accuracy, through a simulation study and through real-world test cases. Unlike existing manual digitization methods, our algorithm firstly automates this process, thereby enhancing efficiency and reducing potential errors. The algorithm is implemented in an R package and an R Shiny for ease of use and is also publicly available on Github. The accuracy of automated digitization is expected to simplify and streamline extraction of information from KM plots that simplifies the conduct of health technology assessments and meta-analyses.

We designed our algorithm as a dynamic tool that can be enhanced over time with new features, either by the authors or the open-source community. Despite its strengths, the automated digitization algorithm has limitations. Although it outperformed manual digitization in the majority of instances in our simulation study, there are edge cases where manual digitization might be preferred in the real-world setting.

Input image format and quality can impact the algorithm’s performance during various digitization stages. The algorithm requires the user to clean the image manually before running the digitization. For example, the detection of axes relies on a clear background, and some KM plots have background grids composed of black lines, which will lead to a failure in process curve pixels. Also, the performance of the OCR technology used in digitization is affected by the input image quality, as it depends on its ability to identify unexpected word descriptions like survival analysis results or p-values for testing treatment effect differences. The automated digitization relies on the input image having perfect rotational registration. If rotation occurs, which is mostly the case with scanned KM plots from hard copies of scientific papers or pictures captured by phone cameras, the algorithm may fail to identify the axes and curves.. For KM plots with multiple curves (groups), we distinguish curves by colors. However, the algorithm cannot distinguish multiple curves with the same color and hue on a single plot, which is a special case. In real-world scenarios, most plotting engines will differentiate colors if multiple patient curves exist. We acknowledge that it is good practice to plot KM curves for different groups in distinct colors or hues, such as red and blue or black and gray for two curves, to help readers visually compare the survival conditions between patient groups. Finally,our algorithm attempts to remove censoring markers that are a different color than the curves, but in more complex cases it can become difficult to distinguish between censoring markers and individual censoring times. Thus, improving image quality and checking image format before digitization is recommended.

Another scenario where automated digitization may perform relatively poorer is when multiple curves are presented on the same plot or when there is considerable overlap between survival curves. The current method of separating or imputing missing curves due to overlap relies on the user indicating how many curves are present in an image. Additionally, the presence of censoring markers complicates KM curve digitization, especially since censoring is represented in different forms across different software.

The primary aim of SurvdigitizeR is to facilitate the extraction of survival probabilities from Kaplan–Meier (KM) plots. In the method ‘Reproduce Individual Level Survival Data’ section, we provide an approach to reproduce IPD using the survival probability output of our SurvdigitizeR and the survHE R package. This method currently applies only under conditions where the risk set size is known, which is often reported at the KM plot. In future versions of SurvdigitizeR, we aim to integrate IPD reproduction and survival analysis. Potential additions to our current SurvdigitizeR could include detecting confidence intervals or confidence bounds and improving the detection of censoring markers on the graph, thereby significantly enhancing the translation of digitized points into IPD.

As we aim to enhance the capabilities of SurvdigitizeR, a promising extension involves adapting the algorithm to automatically digitize and analyze cumulative incidence curves, which estimate the probability of specific events occurring within a defined time period [13, 14]. Unlike KM curves that start at time zero with a probability of one and typically descend over time to indicate survival probabilities, cumulative incidence curves often start at the origin and ascend, reflecting the accumulating risk of a specific event in the presence of competing risks. This difference in trajectory—KM curves moving downward to the right, and cumulative incidence curves moving upward—highlights the distinct nature of the data each represents. Additionally, while KM curves present survival probabilities ranging from 0 to 1, cumulative incidence curves provide direct probabilities of events, requiring careful handling of the y-axis, which usually ranges from 0 to the highest observed or estimated event probability, often less than 1. To effectively adapt our algorithm for these curves, we will need to develop methods to distinguish between different types of events, especially in settings with competing risks. This will likely involve advanced image processing techniques capable of identifying various line styles or markers.

The algorithm is currently hosted as an open-source script on an online repository https://github.com/Pechli-Lab/SurvdigitizeR. Although users are expected to have a basic understanding of R to modify the code of the algorithm, we have also developed a user-friendly web-based application using R Shiny to help users navigate the algorithm even without knowledge of R.

## Conclusion

In conclusion, the open-source algorithm SurvdigitizeR automates the digitization process and requires minimal user interaction. It has been shown to be accurate in digitizing KM curves in both simulated and real-world situations, displaying small bias and close agreement with traditional manual digitization methods. Although the algorithm has its limitations, its dynamic nature allows for continuous improvements and adaptations. By making it accessible via a user-friendly web-based application, we hope to enable broader usage and invite collective enhancements from the open-source community. Future work will focus on refining the algorithm based on real-world testing and adding new features to increase the algorithm’s functionality.

## Methods

### SurvdigitizeR algorithm

This section offers a technical overview of the methods employed to develop the algorithm for digitizing published KM curves. At the same time, this section delves into the technical aspects of the algorithm.

The digitization process encompasses several stages, each of which is detailed below:

#### Step 0: Pre-processing

Prior to running the digitization algorithm, the user must pre-specify a number of input parameters specific to the curve, such as the number of curves on a figure, or the distance between two consecutive tick marks on the y and x-axis, which also refer to the step size of the breaks on axes. In addition, any legends or free text overlayed on the plot would need to be cleared. Additionally, the digitizer algorithm allows for modification of some input parameters such as sensitivity to background distortion which have a default value but sometimes modifications to their values result in improvements to the digitization.

#### Step 1: Reading in KM plot

The algorithm can read KM plots saved as.jpeg or.png images. Internally, image data are converted to the Hue-Saturation-Lightness (HSL) colour-space and stored in a matrix based on pixel x and y values. The size of image data matrix is determined by input image resolution that a good input image quality is suggested. The algorithm is equally adept at handling grayscale images by setting Hue and Saturation to zero, using on the lightness component to derive data.

#### Step 2: Detecting plot axes

**Fig. 5:**
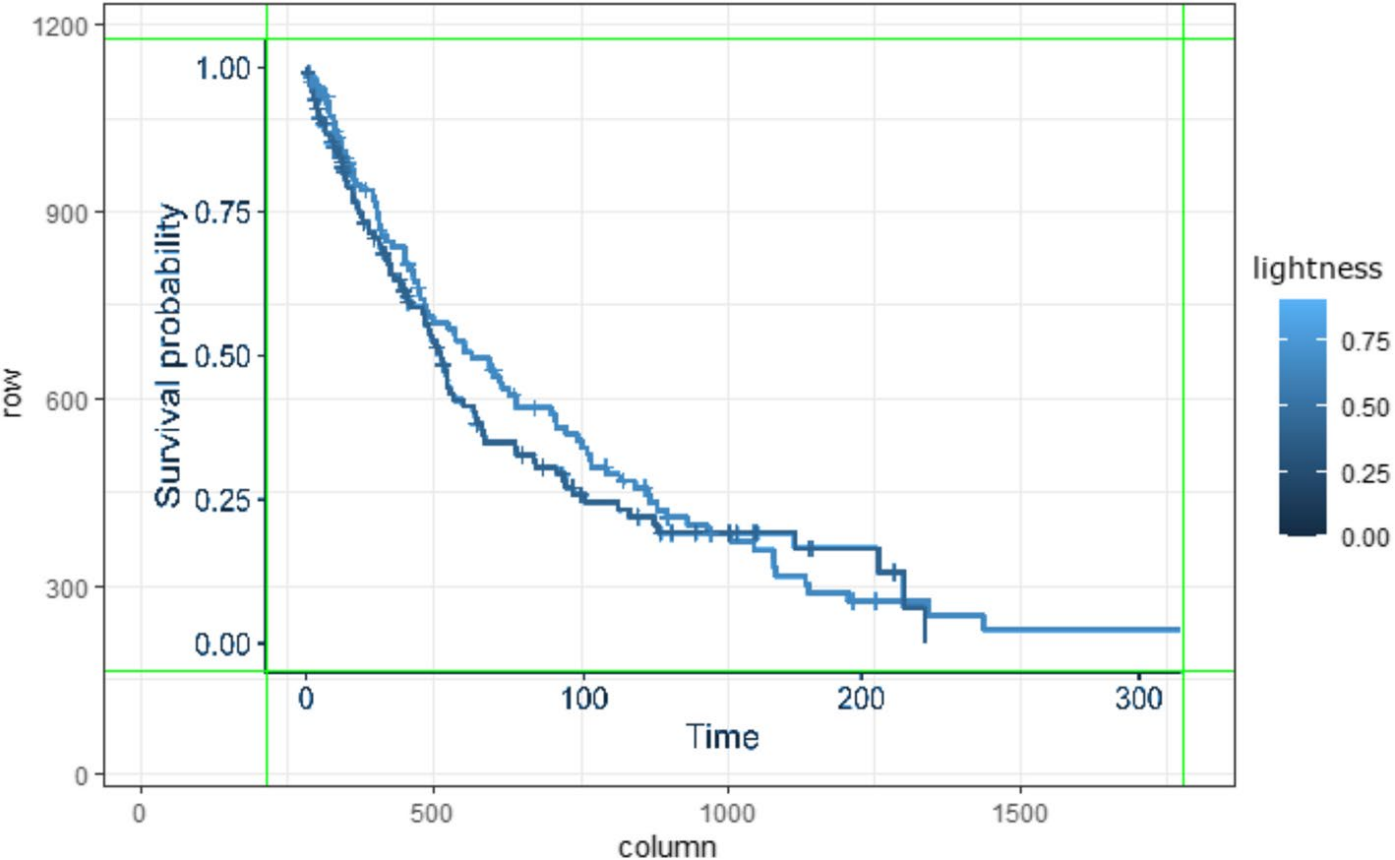
Automatically detected x and y axes’ boundaries

In this step, the algorithm detects the extent of axis lines/borders in the plot based on the number of black pixels in each row and column of the matrix. This process involves calculating the total number of black pixels along each dimension to pinpoint the primary axes. This approach exploits the contrast between the plot elements and the background, allowing the algorithm to identify areas with denser black pixels that typically indicate the presence of x and y axes. Once detected, these axes are isolated to define the active plot area, focusing subsequent analysis solely within these boundaries and excluding any irrelevant peripheral content. An illustration of this can be seen in Fig. 5 and [9]. For subsequent steps, the algorithm only considers the image content within these boundaries, ignoring everything outside, with the exception of step 6 – range detection.

#### Step 3: Cleaning plot background and legend/line labels

In the HSL colour space, the lightness property significantly simplifies the process of detecting colours close to white: pixels with a lightness value greater than or equal to 0.8 will be considered white. Conversely, pixels with a lightness value less than or equal to 0.2 will be black or dark gray, while visibly colourful pixels will fall within the remaining range (0.2, 0.8).

Considering this, eliminating white background pixels or very light gray grid-line pixels is relatively simple, requiring the filtering out of colours with a lightness value ≥ 0.8. To remove any remaining text on the image, we employ R’s Tesseract Optical Character Recognition (OCR) engine [15]. Since the text is typically black, we run an OCR task overall black pixels in the image. If any are recognized as words, we remove them.

**Fig. 6:**
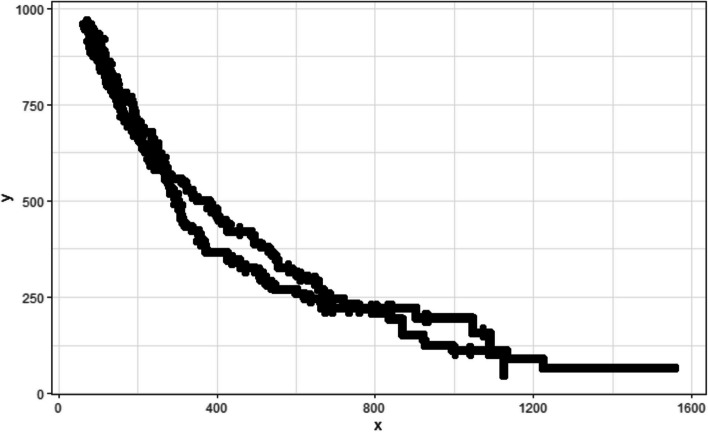
Curve pixels remaining after removing the background

The remaining pixels are stored in a matrix, which only contains pixels associated with a KM curve. Figure 6 shows the result of this step, with all remaining pixels (regardless of colour) marked as black. The axes, generated by the R ggplot package, illustrate that the curve pixel’s coordinate values are: x-axis from 0 to 1600 pixels and y-axis from 0 to 1000 pixels.

#### Step 4: Colour-based curve detection

**Fig. 7:**
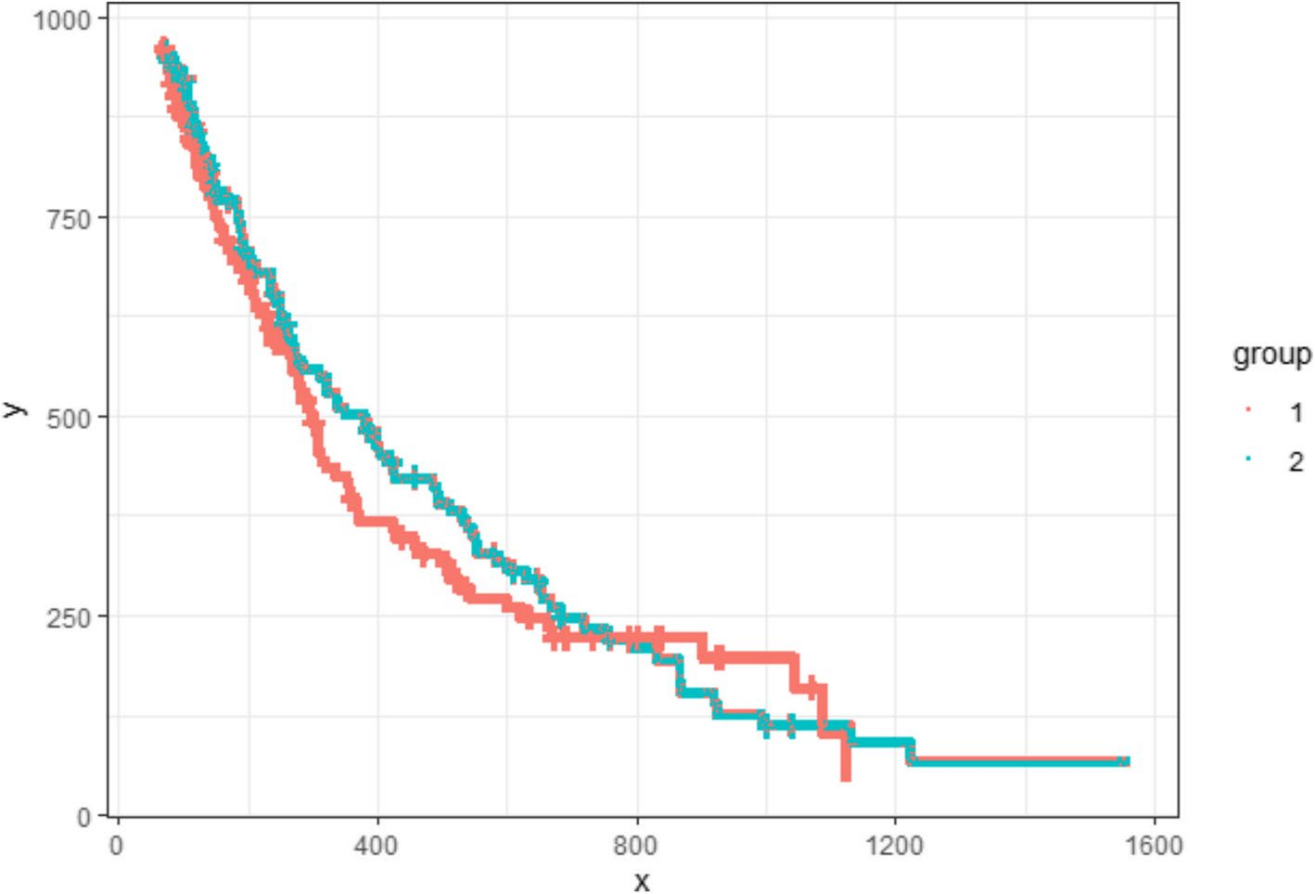
Pixels grouped by their colour

In this step, we group the remaining pixels based on their colour to approximate the associated KM curves. We utilize the HSL values of every pixel and cluster them using the k-medoids clustering algorithm due to its accuracy, time efficiency, and ability to handle outliers [16]. We provide an option to enhance the hue and lightness components by multiplying the value by a constant factor. The number of clusters is set equal to the number of curves in the graph. We provide an option to remove black censoring mark pixels or lines before clustering in the same manner as the previous step’s background removal by filtering the darkest color. While this method performs reasonably well for most pixels in each curve, it occasionally generates minor artifacts, as illustrated in Fig. 7. The artifacts’ effect on digitization performance will be alleviated in Step 5.

#### Step 5: Overlap and artifact detection

In this step, we aim to remove the artifacts produced in Step 4 and ensuring each curve is represented as a single-pixel wide path, regardless of the initial thickness of the line in the image. In Step 4, we clustered pixels based on their color features using HSL scales. Building on this, we now further assess pixel cluster memberships by assessing their spatial relationships. Specifically, we determine a pixel’s affiliation to a curve by evaluating distances on a plane defined by the x and y corrdinates. The core of this step involves the application of a distance-weighted k-nearest-neighbor (k-NN) similarity metric to evaluate how ‘well-clustered’ each pixel is within its group [17]. Specifically, for a pixel point $p$ with nearest neighbors $N{N}_{p}=\left\{{n}_{1},{n}_{2},\dots \right\}$ ordered by ascending distance to $p$ (i.e., $dist\left(p,{n}_{1}\right)\le dist\left(p,{n}_{2}\right)\le \dots$), we define its *knn* metric as:

$$knn=\sum_{i=1}^{k}\frac{comp\left(p,{n}_{i}\right)}{dist(p,{n}_{i}{)}^{2}}$$

where *k* is the number of nearest neighbors examined (by default we set k to 20), $dist\left(p,{n}_{i}\right)$ is the Euclidean distance between $p$ and ${n}_{i}$ and:

$$comp\left(p,{n}_{i}\right)=\left\{\begin{array}{c} \begin{array}{cc}1,& \text{if }group\left(p\right)=group\left({n}_{i}\right)\end{array}\\ \begin{array}{cc} -1,& {\text{otherwise}}\end{array}\end{array}\right.$$

**Fig. 8:**
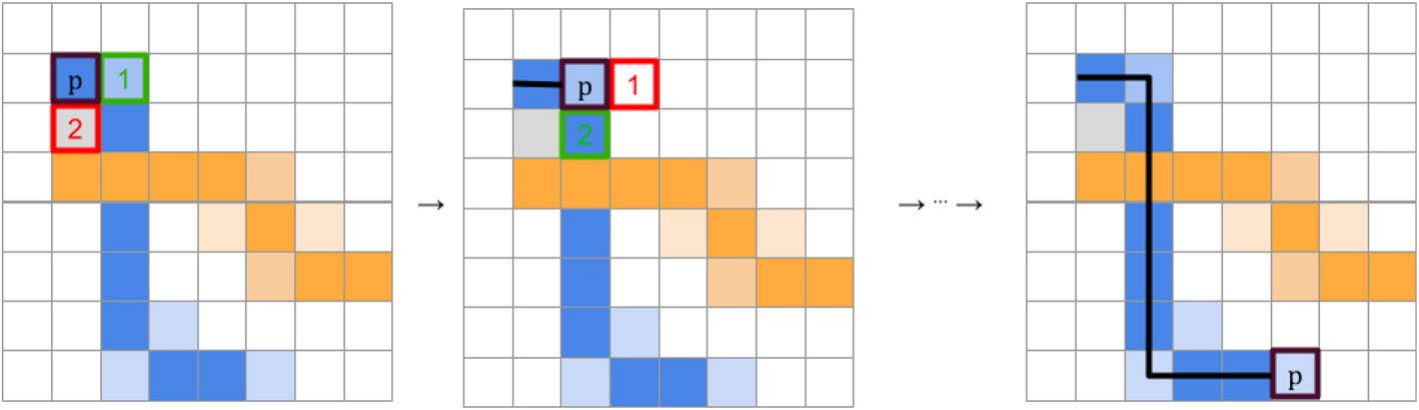
Step-by-step illustration of the artifact removal process

**Fig. 9:**
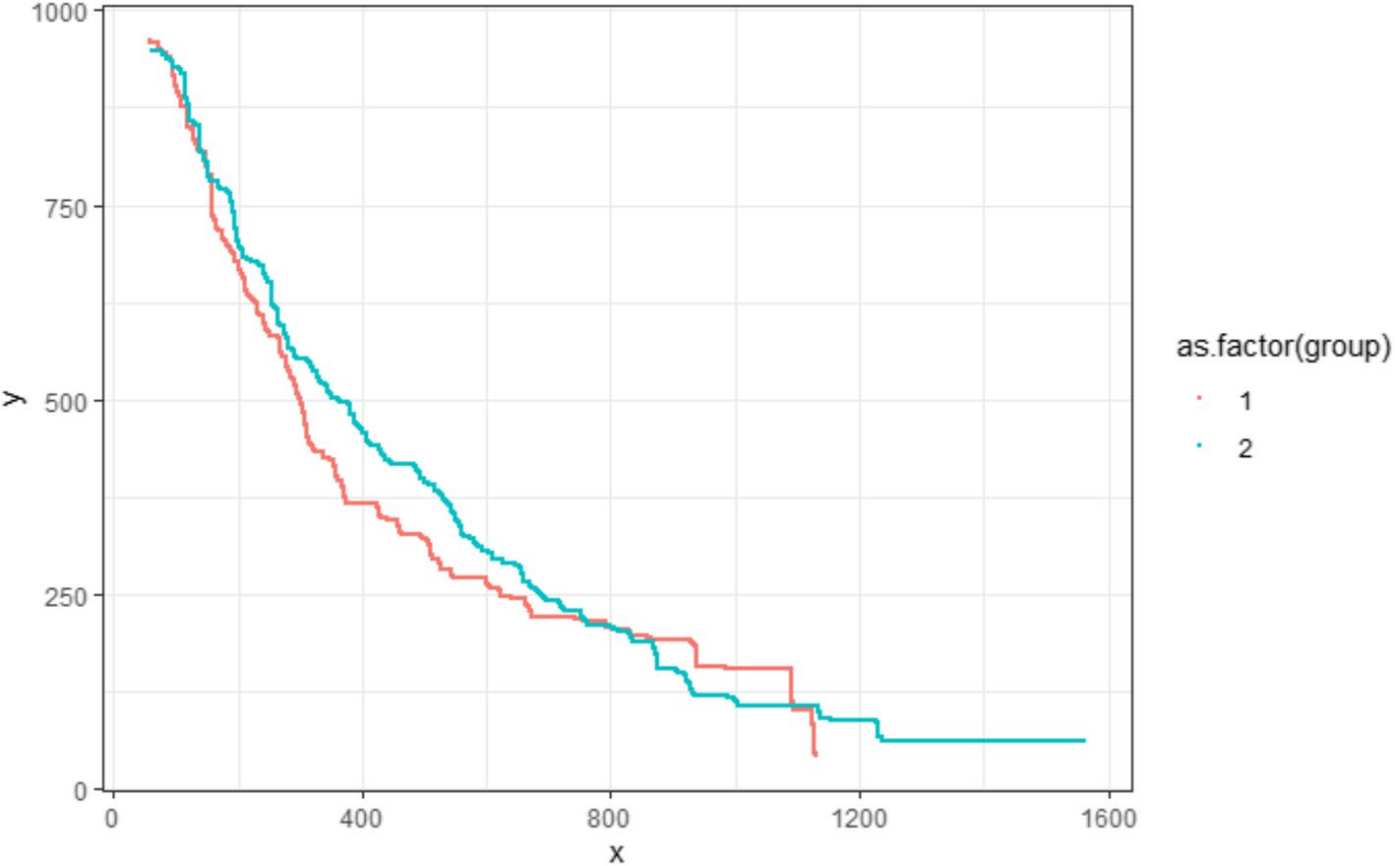
Resulting single-pixel-wide curve paths

This metric aids in determining how tightly a pixel is integrated within its cluster. With this metric calculated for every remaining pixel in the image, we proceed to identify a single-pixel wide path to trace each curve, starting at the top-most, left-most point. Due to the decreasing nature of the survival probability curves, as we navigate from left to right (increasing x-values), the algorithm evaluates two potential paths for each consecutive pixels: verticially downwards $\left(x,y\right)\to \left(x, y-1\right)$ or horizontally to the pixel to the right $\left(x,y\right)\to \left(x+1, y\right)$. If both directions offer a colored pixel, the decision on which path to follow is made by comparing *knn* scores and the pixel with the higher score is chosen, which signifies a stronger correlation with the curve’s defined path. This approach ensures that the traced path adheres closely to the most coherent and statistically supported route through the data points. Also, this approach guarantees that missing segments due to overlaps and artifacts will be “filled in” and other artifacts, outliers and noise will be ignored. An illustration of this process can be seen in Fig. 8, and the end result in Fig. 9.

#### Step 6: *Axis* range detection

This step utilizes the surrounding space outside the axes from step 2 and the final computed ranges of each curve from step 5. OCR is used to verify the axis tick labels against user-provided ranges, and the original scale of each axis is determined. By using OCR, the algorithm ensures that the labels on the axes match the expected ranges, confirming the precision of the data points depicted on the plot. Following verification, the algorithm measures the distance between these tick labels—referred to as the step size—in both the original units (such as days or probability) and in pixel values, derived from the image data matrix. This dual measurement allows for precise mapping of pixel values back to the original scales. This process effectively transforms the plot’s visual data in pixel values back into numerical data that accurately reflects the original graphical representation of survival time and probability.

#### Step 7: Results aggregation

Finally, the result is converted to a format readable by the survival function and stored in a matrix with digitized point ID, time, survival probability and curve identifier columns. Each of the above steps is contained in a corresponding function. The complete method can be invoked using the surv_digitize() method, where several parameters, such as the number of curves, sensitivity, etc., are configurable as arguments.

### Reproduce individual level survival data

**Fig. 10:**
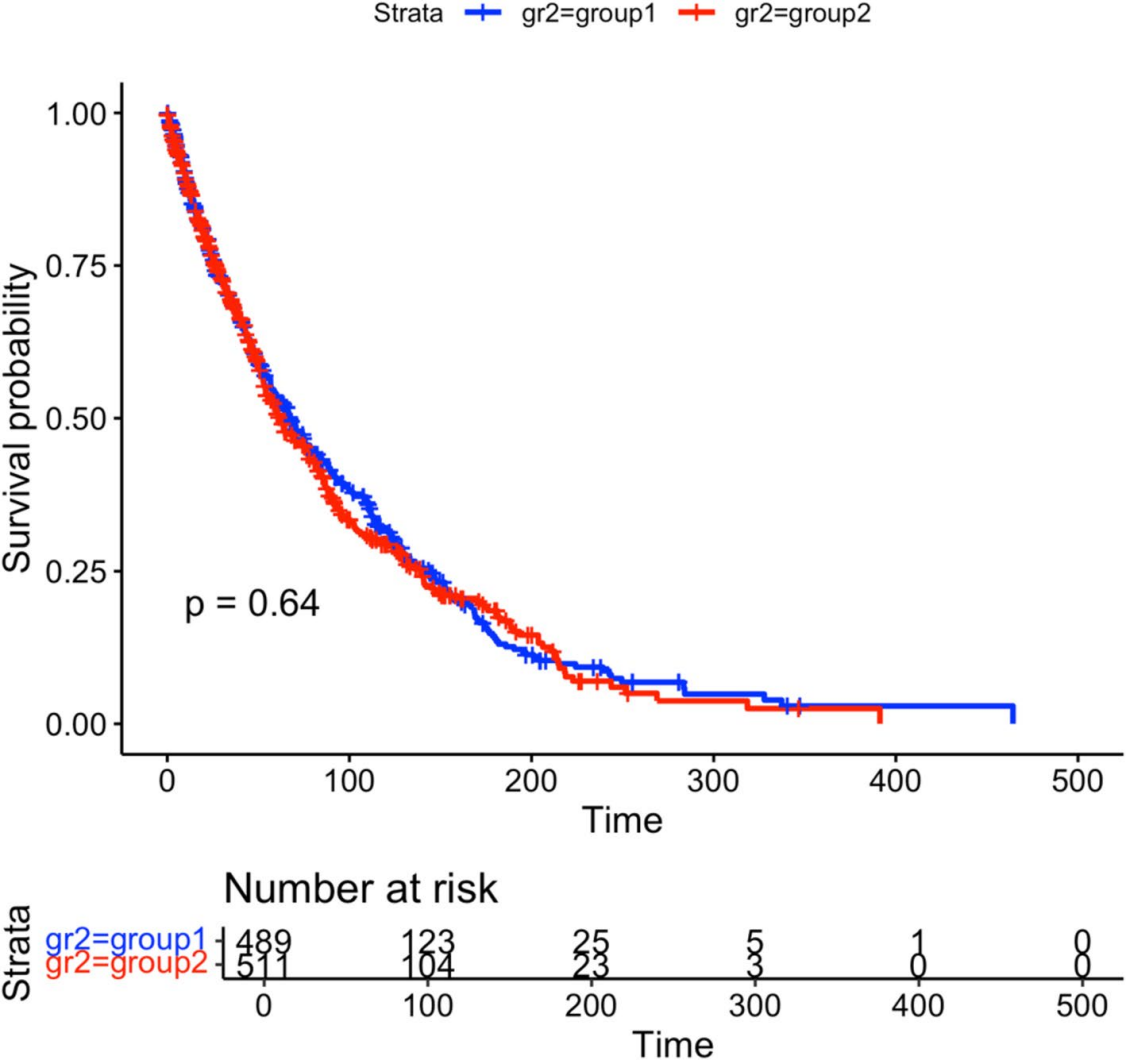
Simulated Plot with 1000 individuals and 2 treatment groups

Baio provided the survHE R package to wrap several tools that perform survival analysis for economic evaluations [18]. This package contains the function digitize() to reproduce individual-level survival data from manual digitized KM curve data through DigitizeIT software [7]. The output of survival probabilities from SurvdigitizeR is compatible with the digitize() function. Together with survHE package, users can automatically construct simulated individual level data for a KM plot with a provided table of number of people at risk, an example shown in Fig. 10.

Firstly, we digitize the survival probabilities for each treatment group using SurvdigitizeR and use the output data table as the digitize() function’s input. Next, we can collect the number of individuals at risk information from the KM plot in Table 3. The input ‘n_risk_inp’ for the digitise() function requires a detailed table in which each digitized survival probability data will be matched to time intervals in Table 3, resulting in Table 4. The columns ‘lower’ and ‘upper’ contain the row indices of the digitized data that fall into the survival times interval. For example, the digitized data for group 1 from rows 1 to 159 corresponds to survival times from 0 to 100.

**Table 3:** Number at risk table for the example figure

Time	Group1	Group2
0	489	511
100	123	104
200	25	23
300	5	3
400	1	0
500	0	0

**Table 4:** Matched digitized data and number at risk table

Interval	lower1	upper1	n_riskgropu1	lower2	upper2	n_riskgropu2
0	1	159	489	1	242	511
100	160	379	123	243	451	104
200	380	439	25	452	561	23
300	440	469	5	562	586	3
400	470	472	1			

**Fig. 11:**
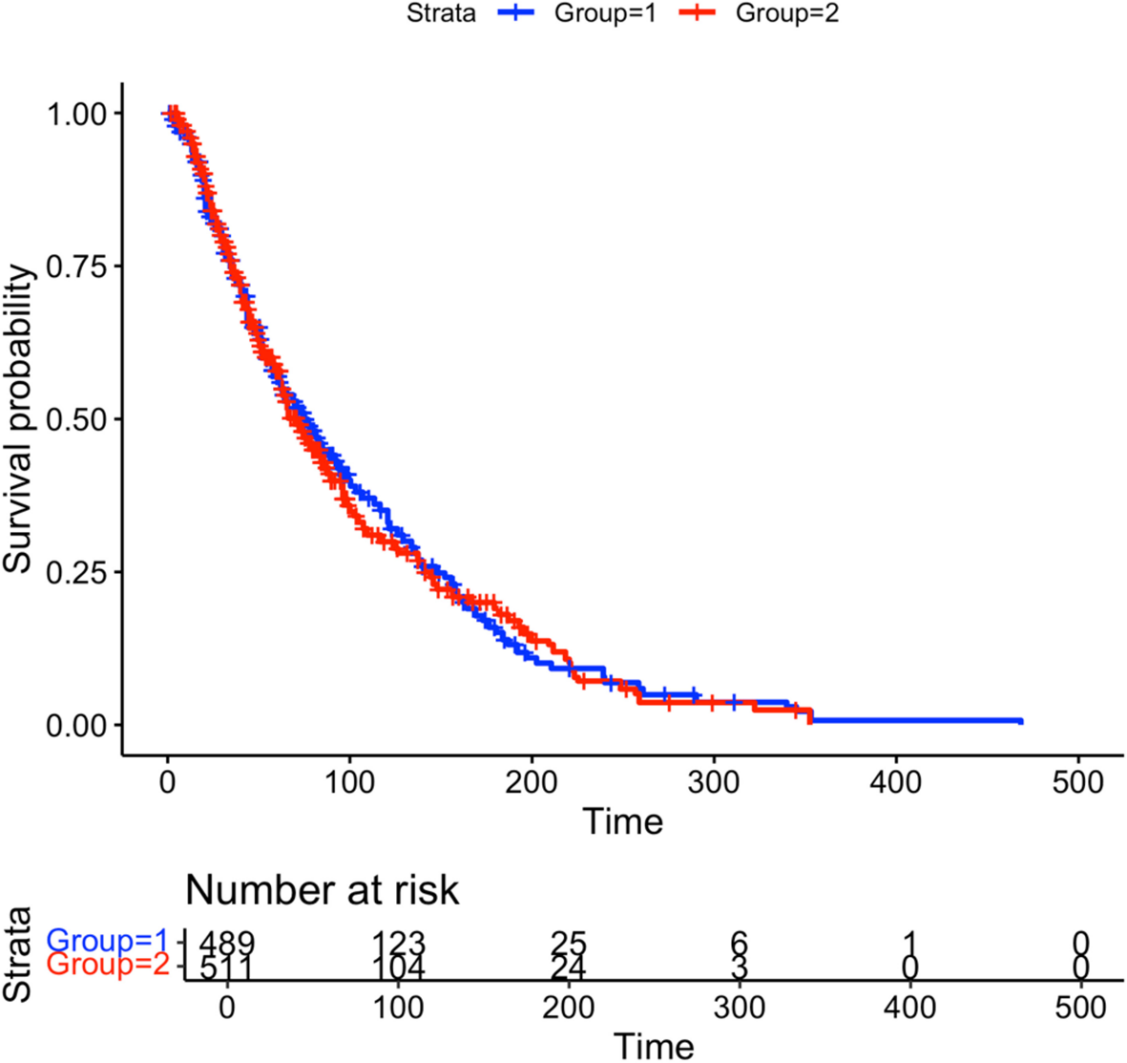
KM Plot of reproduced IPD

Subsequently, the digitise() function will produce the IPD data into “IPDdata.txt”; we can create the KM plot using reproduced IPD as Fig. 11. The number at risk table is exactly the same as the source KM plot, and the estimated event time assigned to individuals are calculated by the digitized survival probabilities and number at risk for each time interval.

### Supplementary Information

Supplementary Material 1: Fig. S1 User Interface for KM Plot Digitization in R Shiny Application.

Supplementary Material 2: Fig. S2 Bland Alman plots illustrating the agreement between manual and automated digitization Test 1 OS curve 1 and curve 2.

Supplementary Material 3: Fig. S3 Bland Alman plots illustrating the agreement between manual and automated digitization Test 1 PFS curve 1 and curve 2.

Supplementary Material 4: Fig. S4 Bland Alman plots illustrating the agreement between manual and automated digitization Test 2 OS curve 1 and curve 2.

Supplementary Material 5: Fig. S5 Bland Alman plots illustrating the agreement between manual and automated digitization Test 2 PFS curve 1 and curve 2.

Supplementary Material 6: Fig. S6 Bland Alman plots illustrating the agreement between manual and automated digitization Test 3 OS curve 1 and curve 2.

Supplementary Material 7: Fig. S7 Bland Alman plots illustrating the agreement between manual and automated digitization Test 3 PFS curve 1 and curve 2.

Supplementary Material 8: Fig. S8 Bland Alman plots illustrating the agreement between manual and automated digitization Test 4 OS curve 1 and curve 2.

Supplementary Material 9: Fig. S9 Bland Alman plots illustrating the agreement between manual and automated digitization Test 4 PFS curve 1 and curve 2.

## Data Availability

The SurvdigitizeR R package for the project can be downloaded at: https://github.com/Pechli-Lab/SurvdigitizeR. The SurvdigitizeR Shiny application can be accessed at: https://pechlilab.shinyapps.io/SurvdigitizeR/.
