# Supplementary figures and images for: SurvdigitizeR: an algorithm for automated survival curve digitization
Source: BMC Med Res Methodol. 2024 Jul 13;24:147. doi: 10.1186/s12874-024-02273-8 (PMC11245803; doi:10.1186/s12874-024-02273-8)

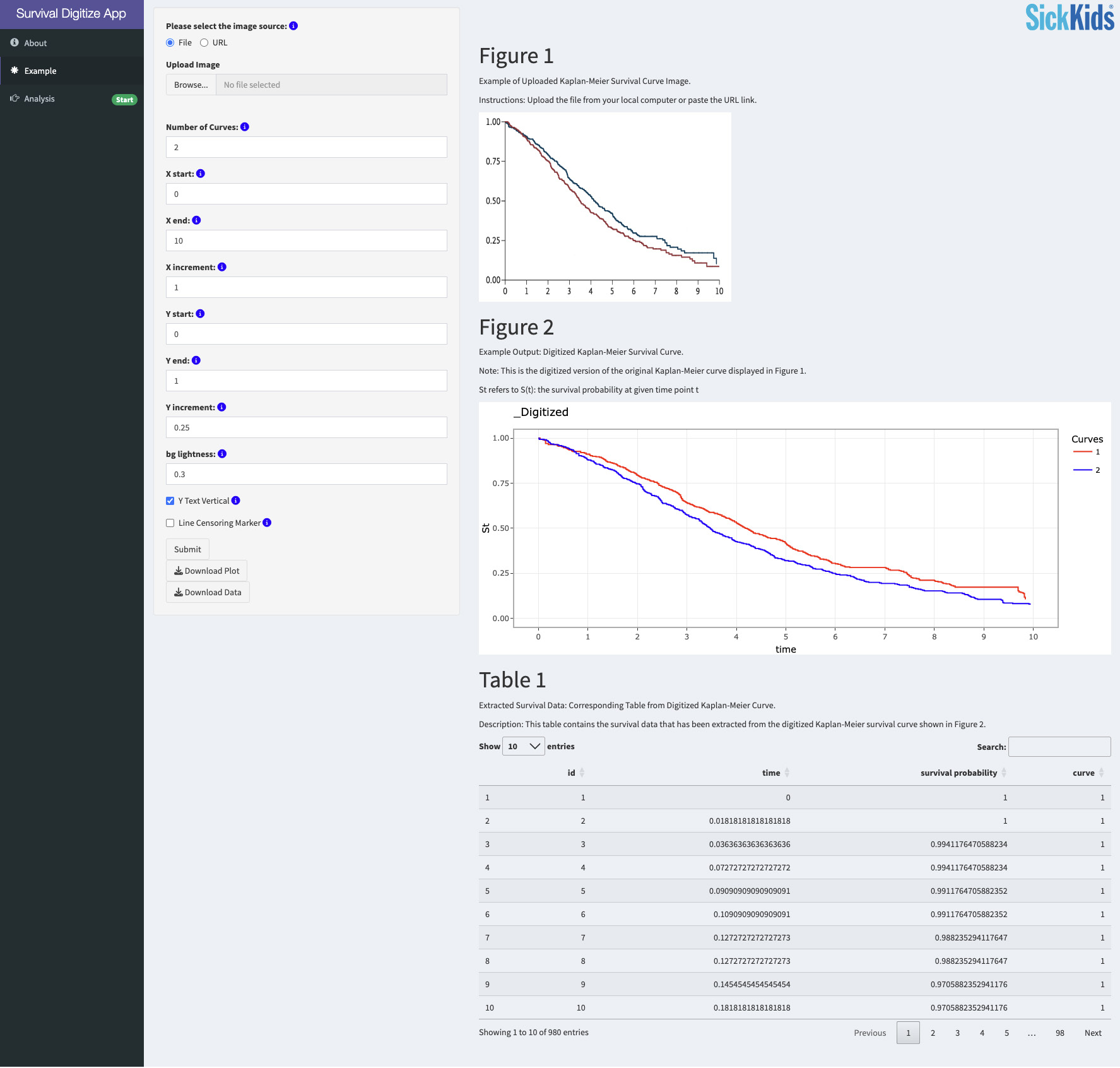

Supplement: Supplementary file 1 — Supplementary Material 1: Fig. S1 User Interface for KM Plot Digitization in R Shiny Application. [file 12874_2024_2273_MOESM1_ESM.jpg]

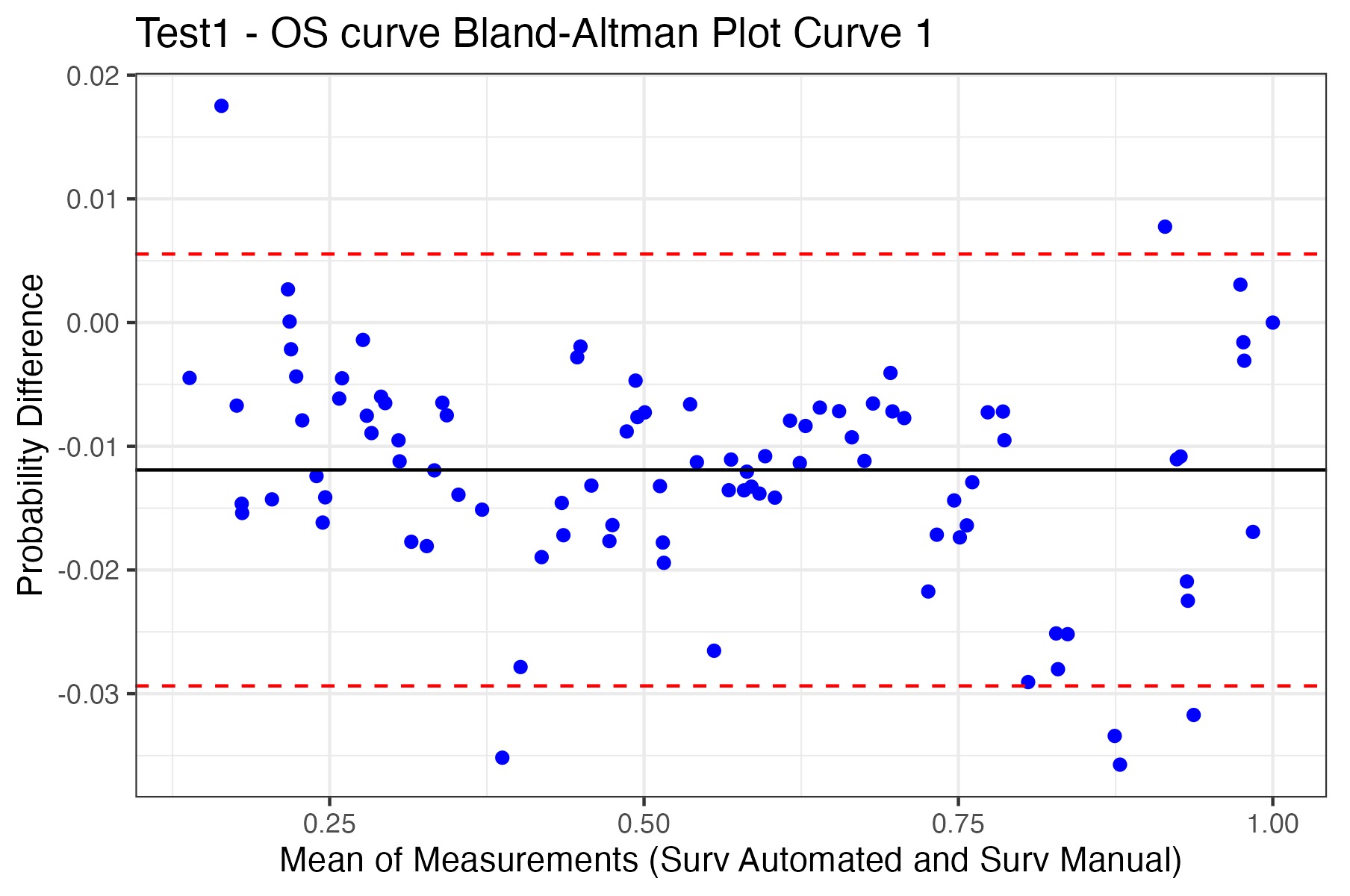

Supplement: Supplementary file 2 — Supplementary Material 2: Fig. S2 Bland Alman plots illustrating the agreement between manual and automated digitization Test 1 OS curve 1 and curve 2. [file 12874_2024_2273_MOESM2_ESM.zip › Figure S2A.jpg]

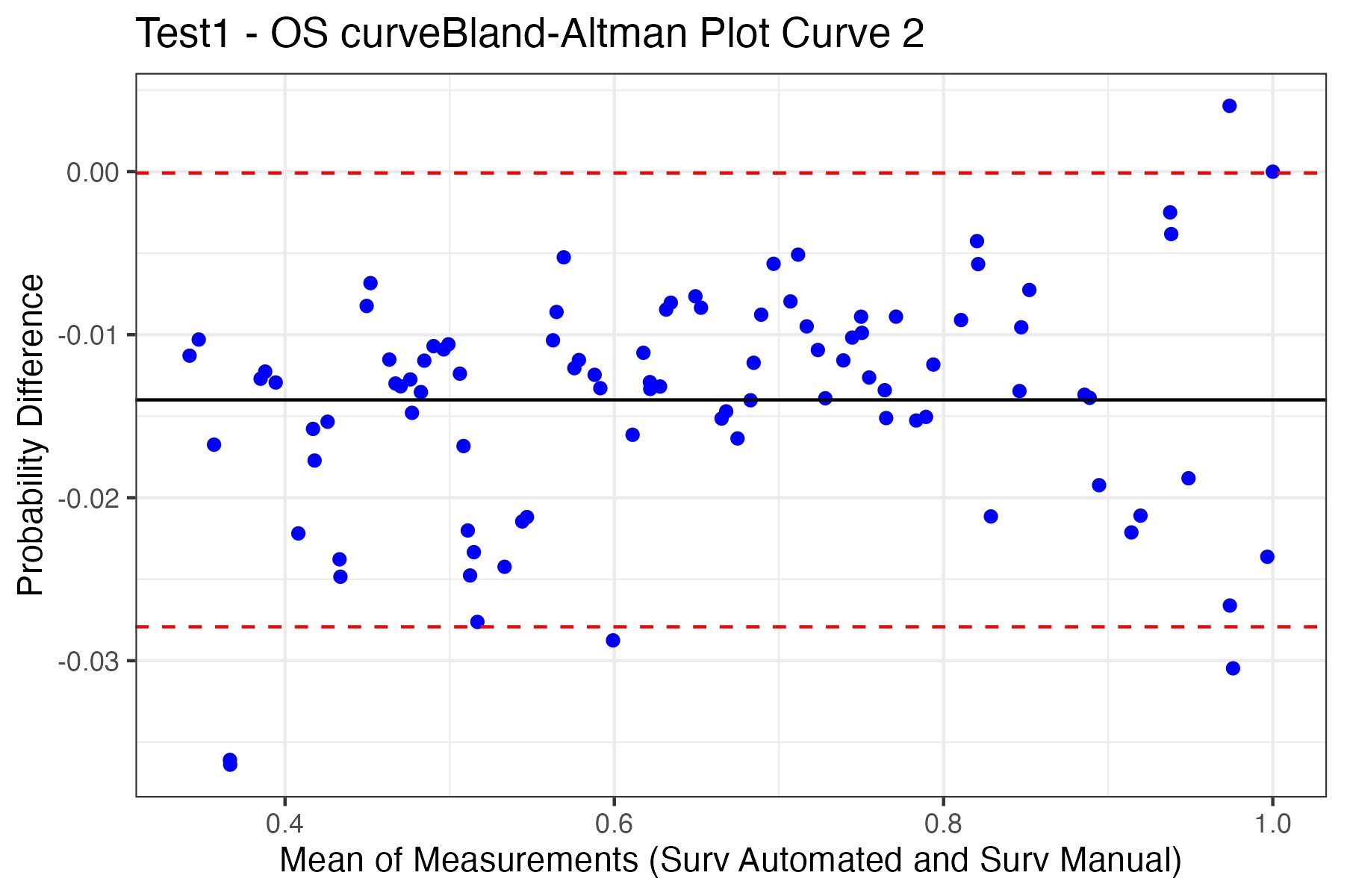

Supplement: Supplementary file 2 — Supplementary Material 2: Fig. S2 Bland Alman plots illustrating the agreement between manual and automated digitization Test 1 OS curve 1 and curve 2. [file 12874_2024_2273_MOESM2_ESM.zip › Figure S2B.jpg]

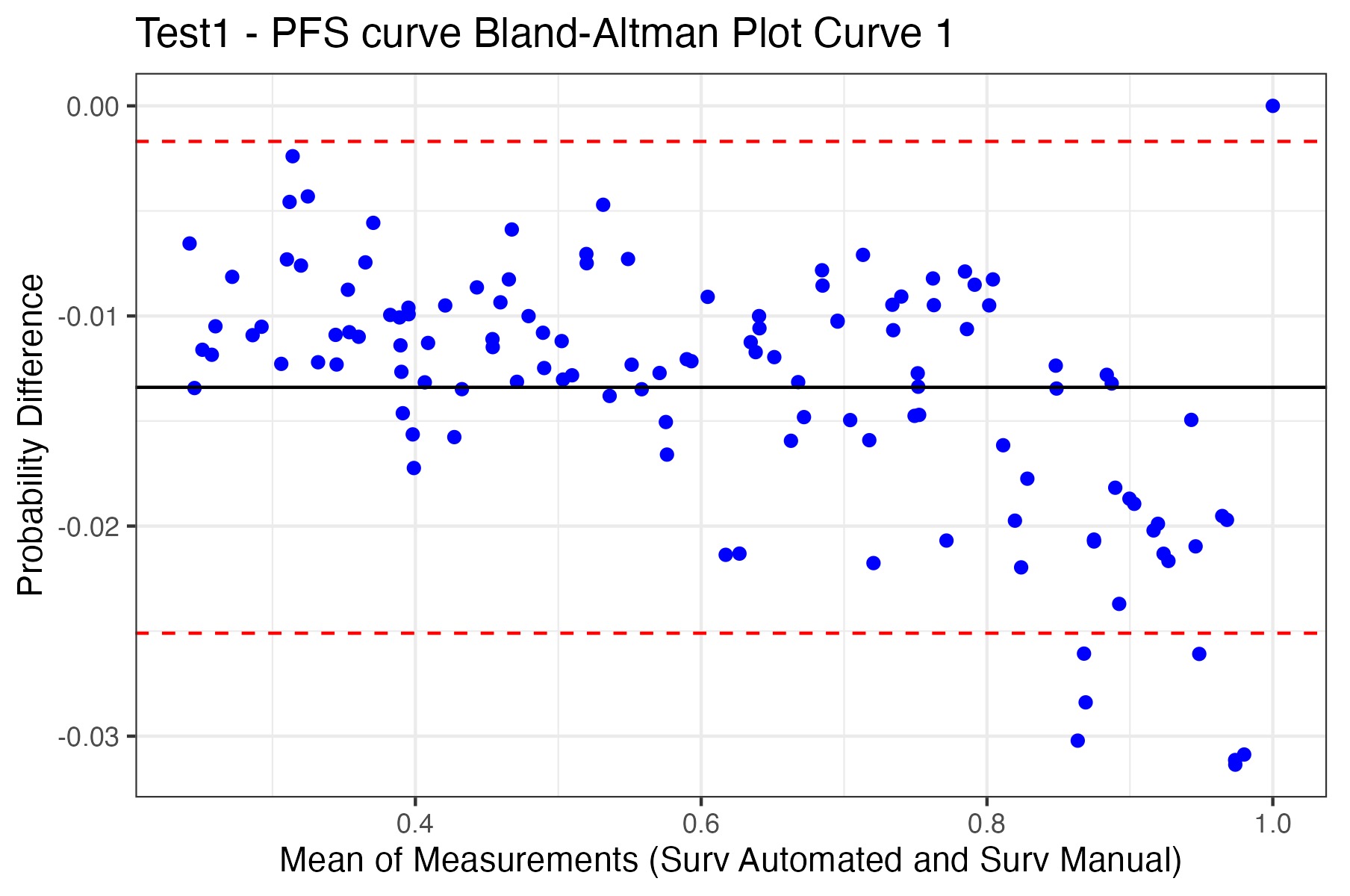

Supplement: Supplementary file 3 — Supplementary Material 3: Fig. S3 Bland Alman plots illustrating the agreement between manual and automated digitization Test 1 PFS curve 1 and curve 2. [file 12874_2024_2273_MOESM3_ESM.zip › Figure S3A.jpg]

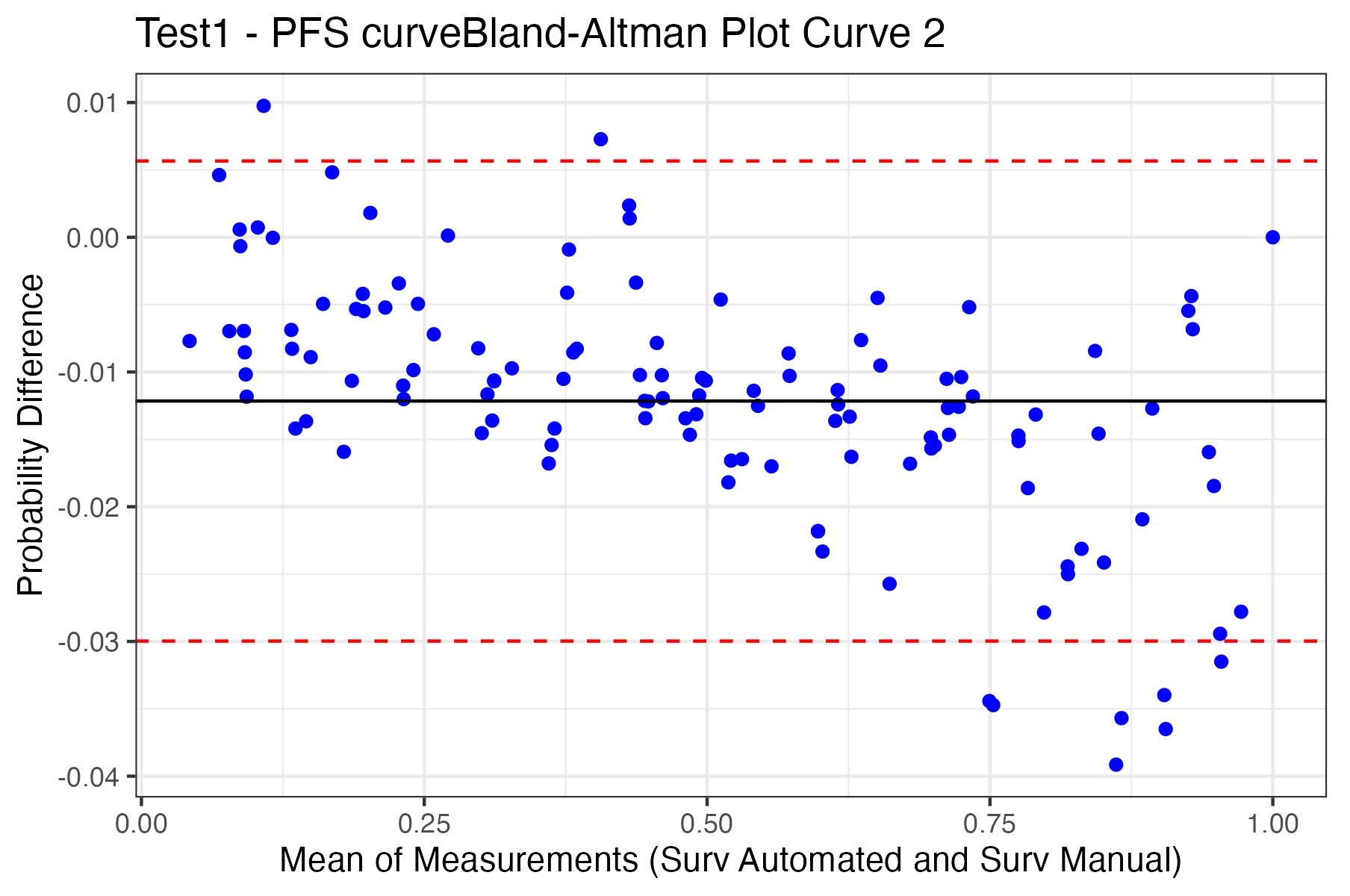

Supplement: Supplementary file 3 — Supplementary Material 3: Fig. S3 Bland Alman plots illustrating the agreement between manual and automated digitization Test 1 PFS curve 1 and curve 2. [file 12874_2024_2273_MOESM3_ESM.zip › Figure S3B.jpg]

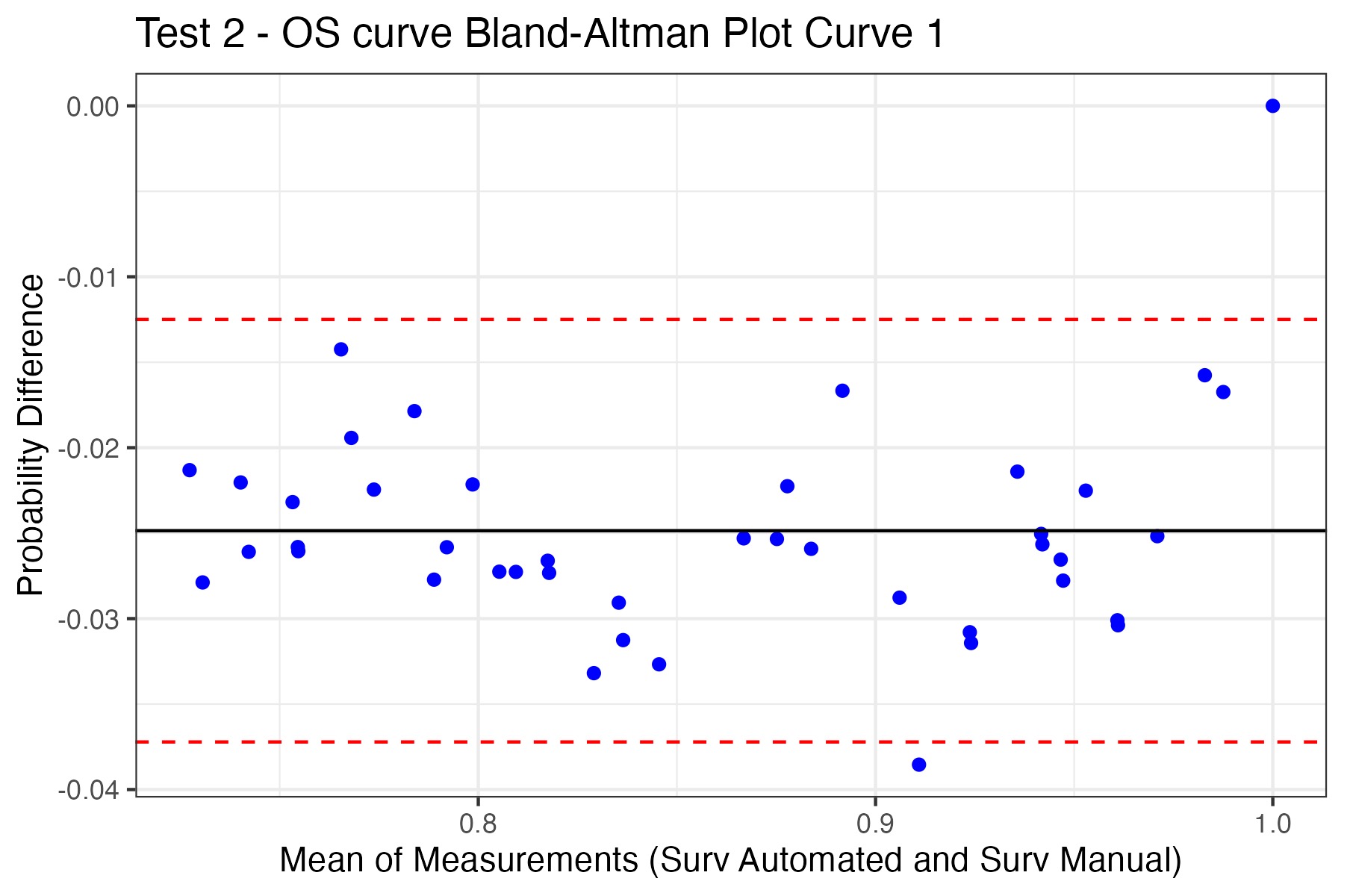

Supplement: Supplementary file 4 — Supplementary Material 4: Fig. S4 Bland Alman plots illustrating the agreement between manual and automated digitization Test 2 OS curve 1 and curve 2. [file 12874_2024_2273_MOESM4_ESM.zip › Figure S4A.jpg]

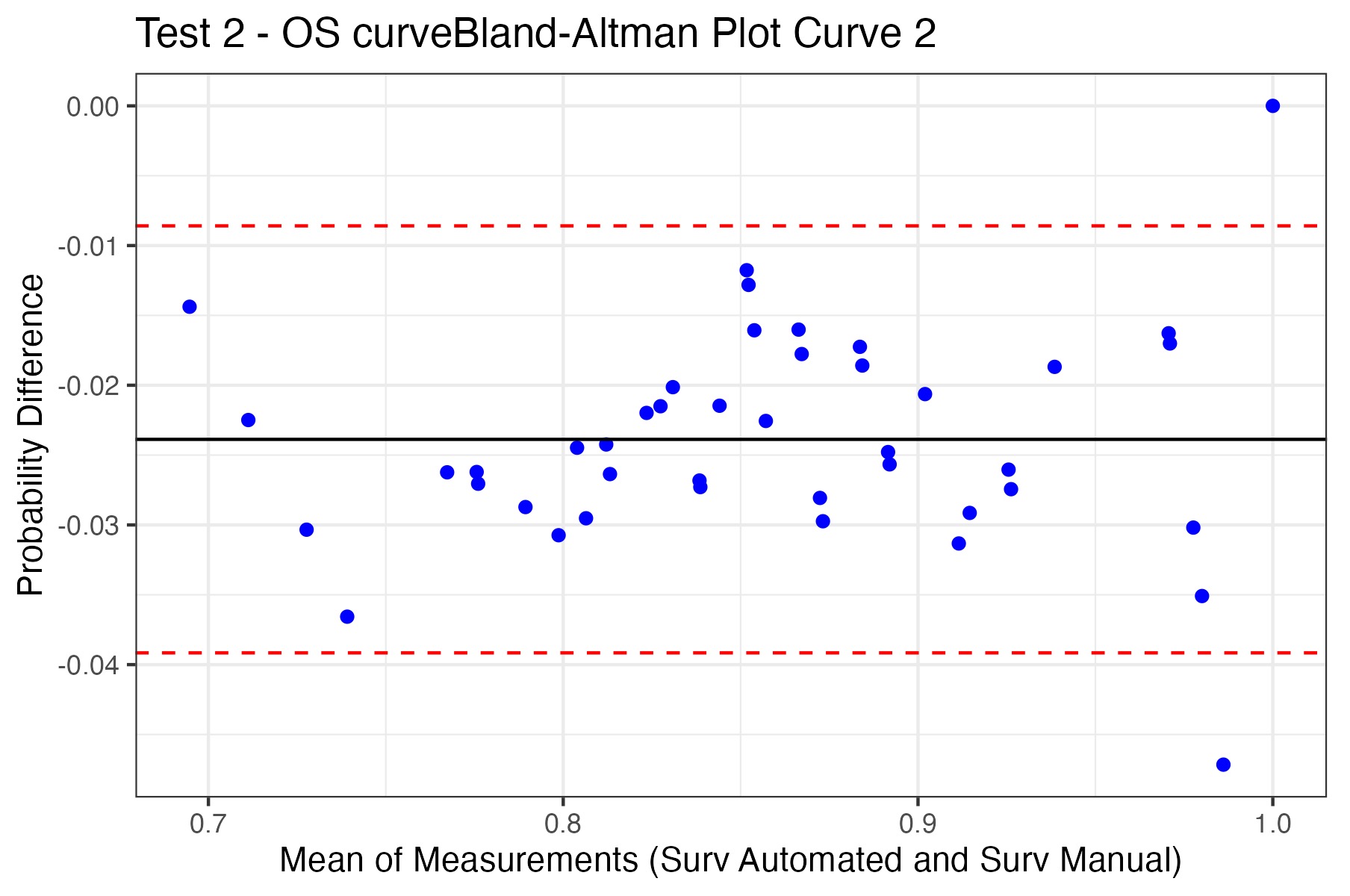

Supplement: Supplementary file 4 — Supplementary Material 4: Fig. S4 Bland Alman plots illustrating the agreement between manual and automated digitization Test 2 OS curve 1 and curve 2. [file 12874_2024_2273_MOESM4_ESM.zip › Figure S4B.jpg]

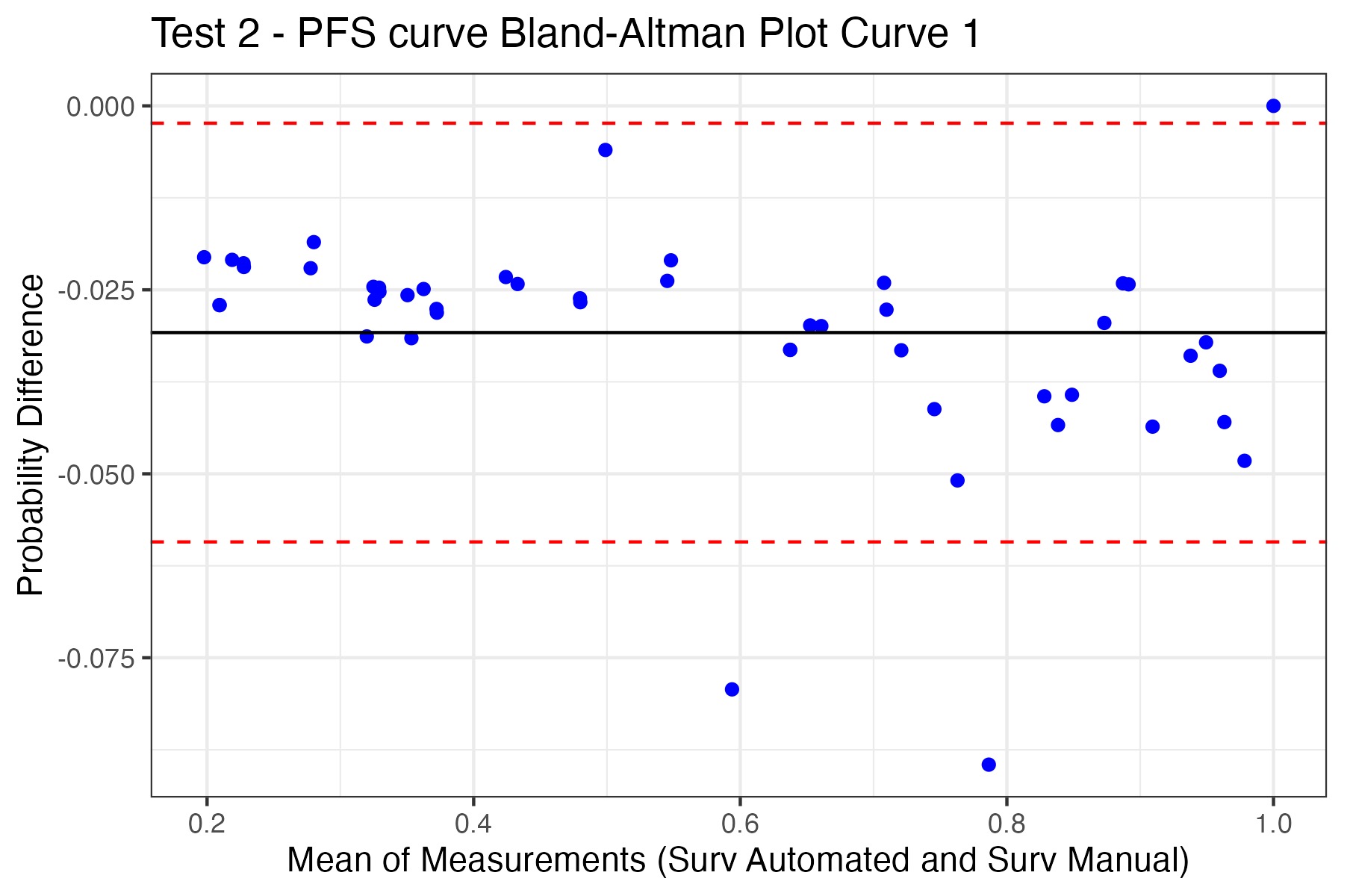

Supplement: Supplementary file 5 — Supplementary Material 5: Fig. S5 Bland Alman plots illustrating the agreement between manual and automated digitization Test 2 PFS curve 1 and curve 2. [file 12874_2024_2273_MOESM5_ESM.zip › Figure S5A.jpg]

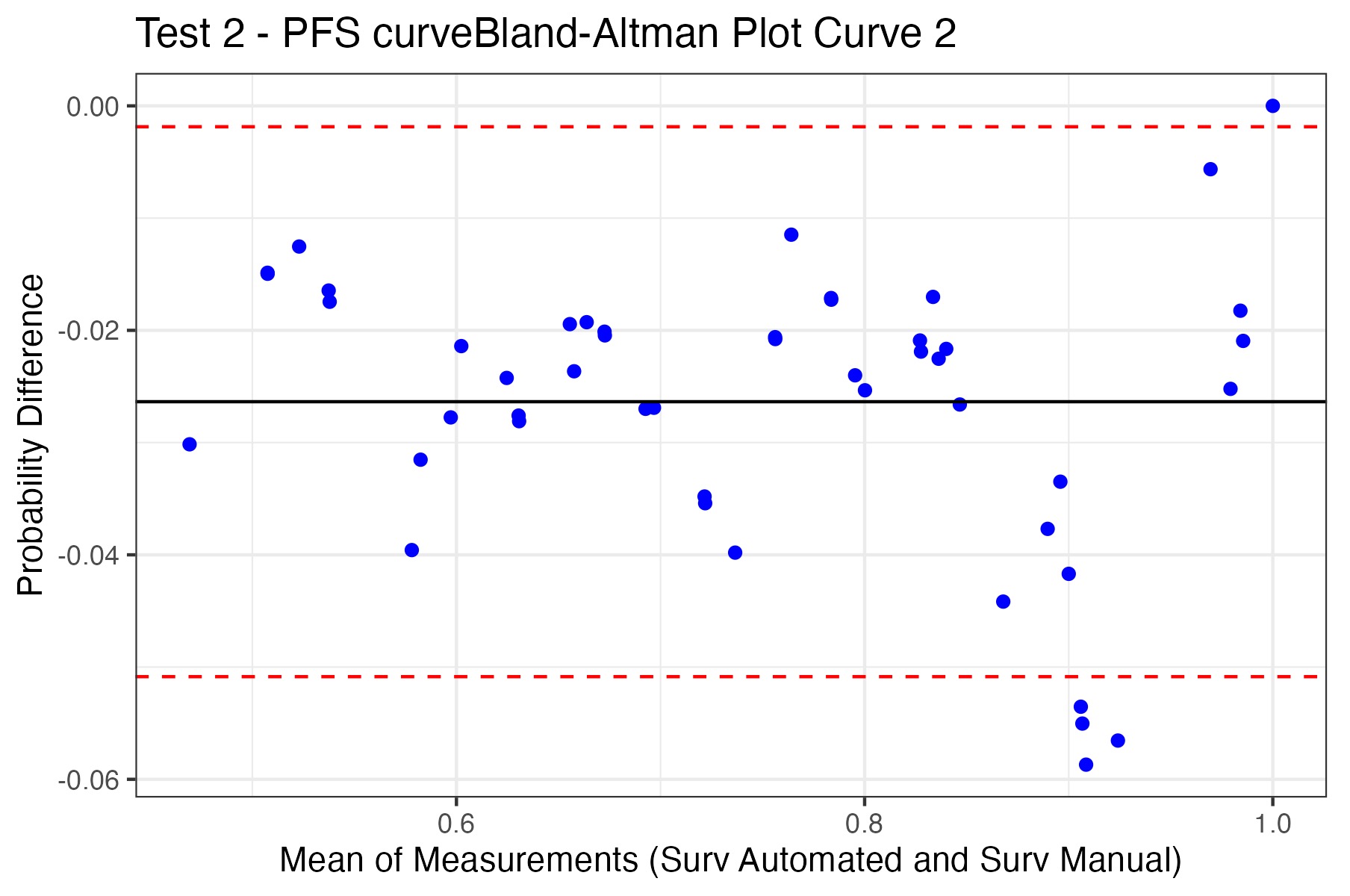

Supplement: Supplementary file 5 — Supplementary Material 5: Fig. S5 Bland Alman plots illustrating the agreement between manual and automated digitization Test 2 PFS curve 1 and curve 2. [file 12874_2024_2273_MOESM5_ESM.zip › Figure S5B.jpg]

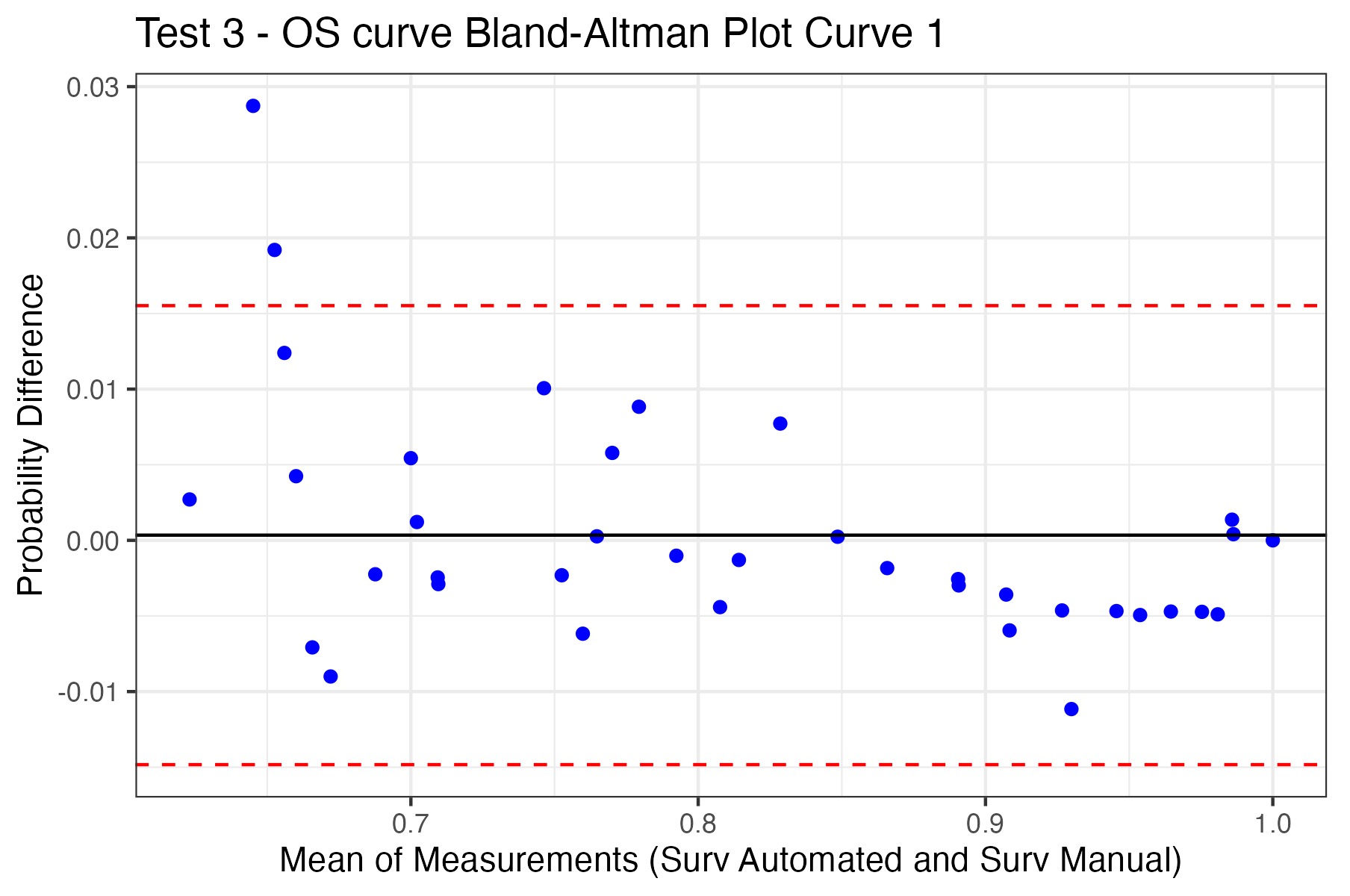

Supplement: Supplementary file 6 — Supplementary Material 6: Fig. S6 Bland Alman plots illustrating the agreement between manual and automated digitization Test 3 OS curve 1 and curve 2. [file 12874_2024_2273_MOESM6_ESM.zip › Figure S6A.jpg]

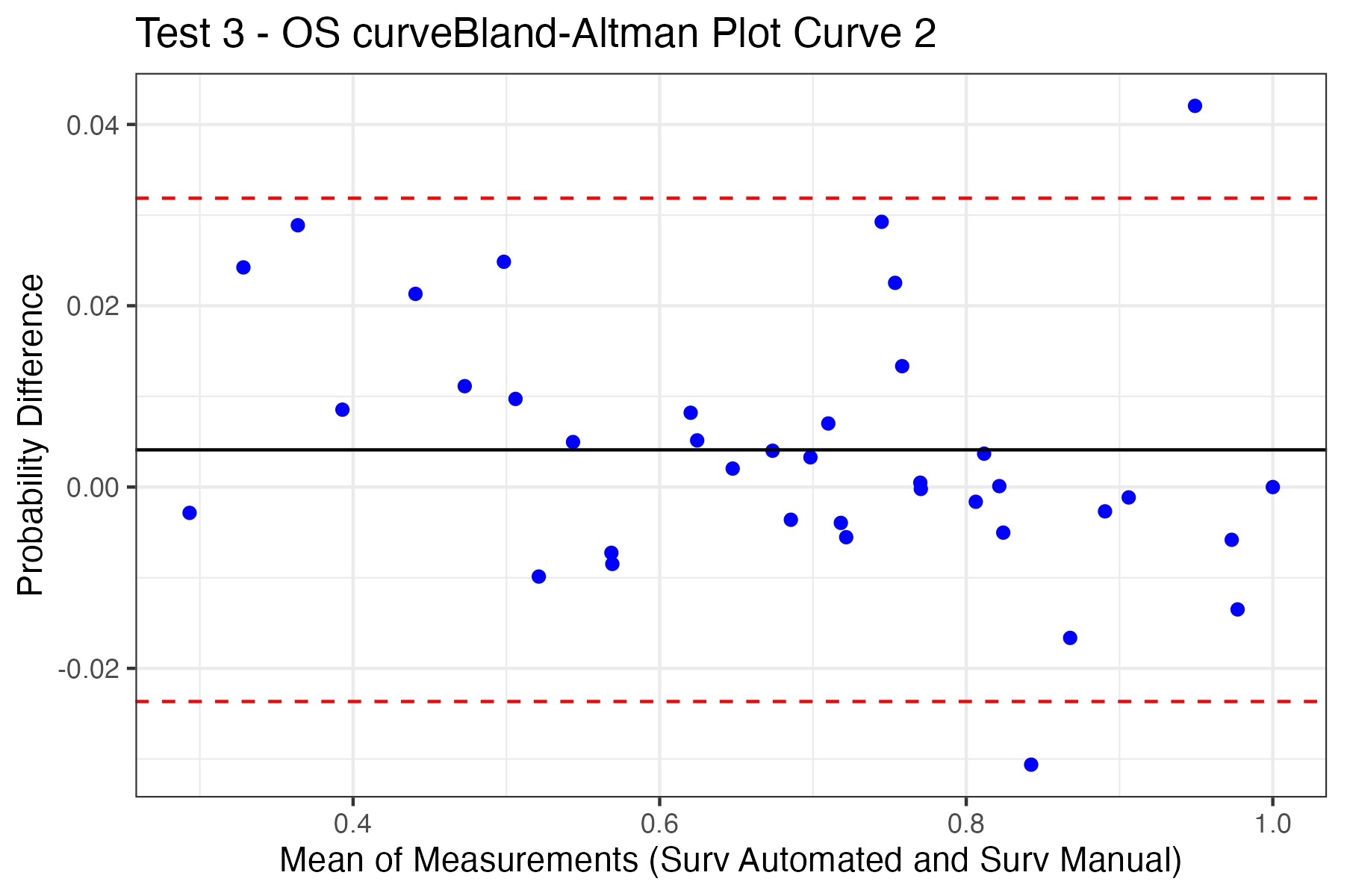

Supplement: Supplementary file 6 — Supplementary Material 6: Fig. S6 Bland Alman plots illustrating the agreement between manual and automated digitization Test 3 OS curve 1 and curve 2. [file 12874_2024_2273_MOESM6_ESM.zip › Figure S6B.jpg]

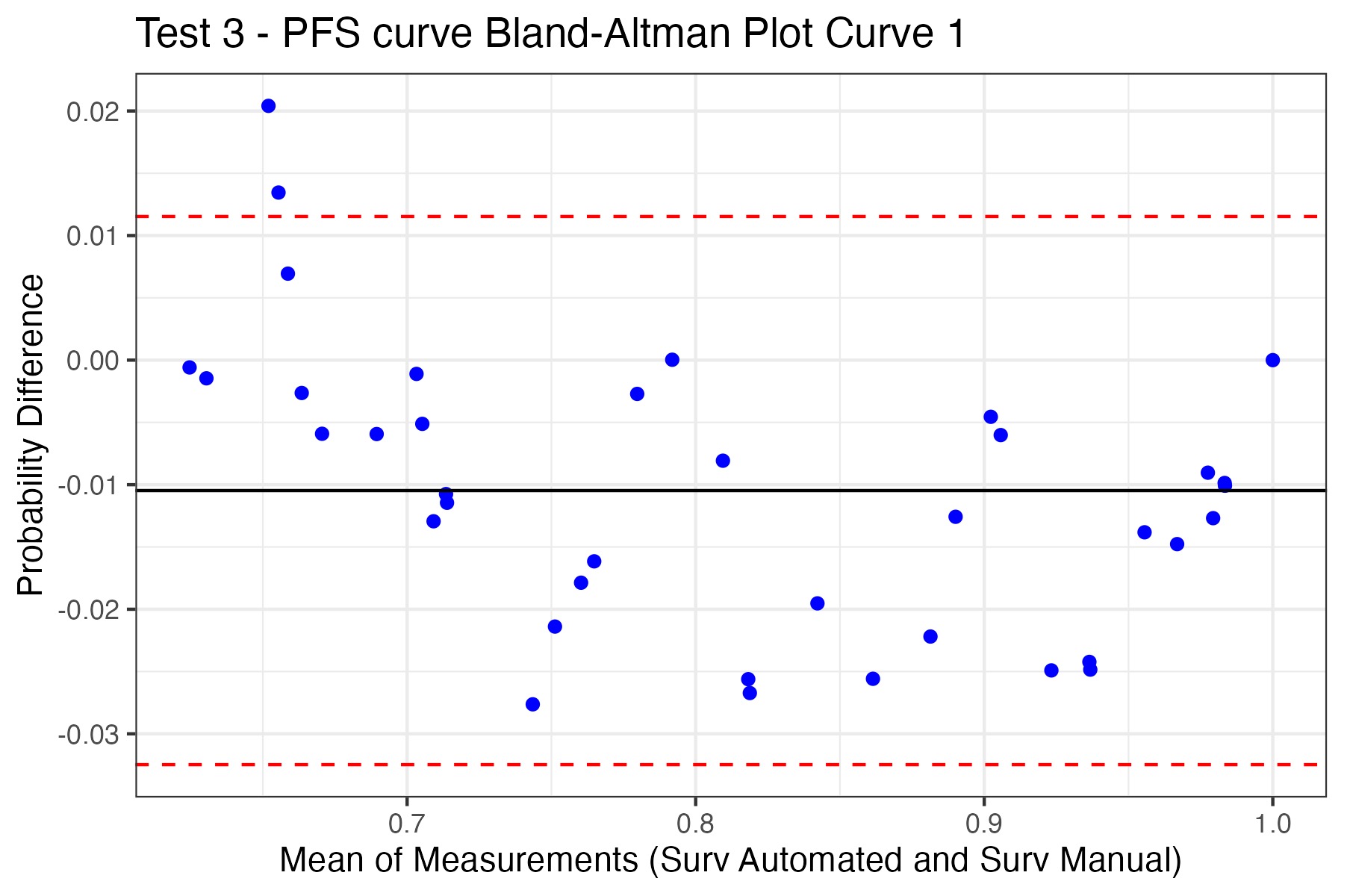

Supplement: Supplementary file 7 — Supplementary Material 7: Fig. S7 Bland Alman plots illustrating the agreement between manual and automated digitization Test 3 PFS curve 1 and curve 2. [file 12874_2024_2273_MOESM7_ESM.zip › Figure S7A.jpg]

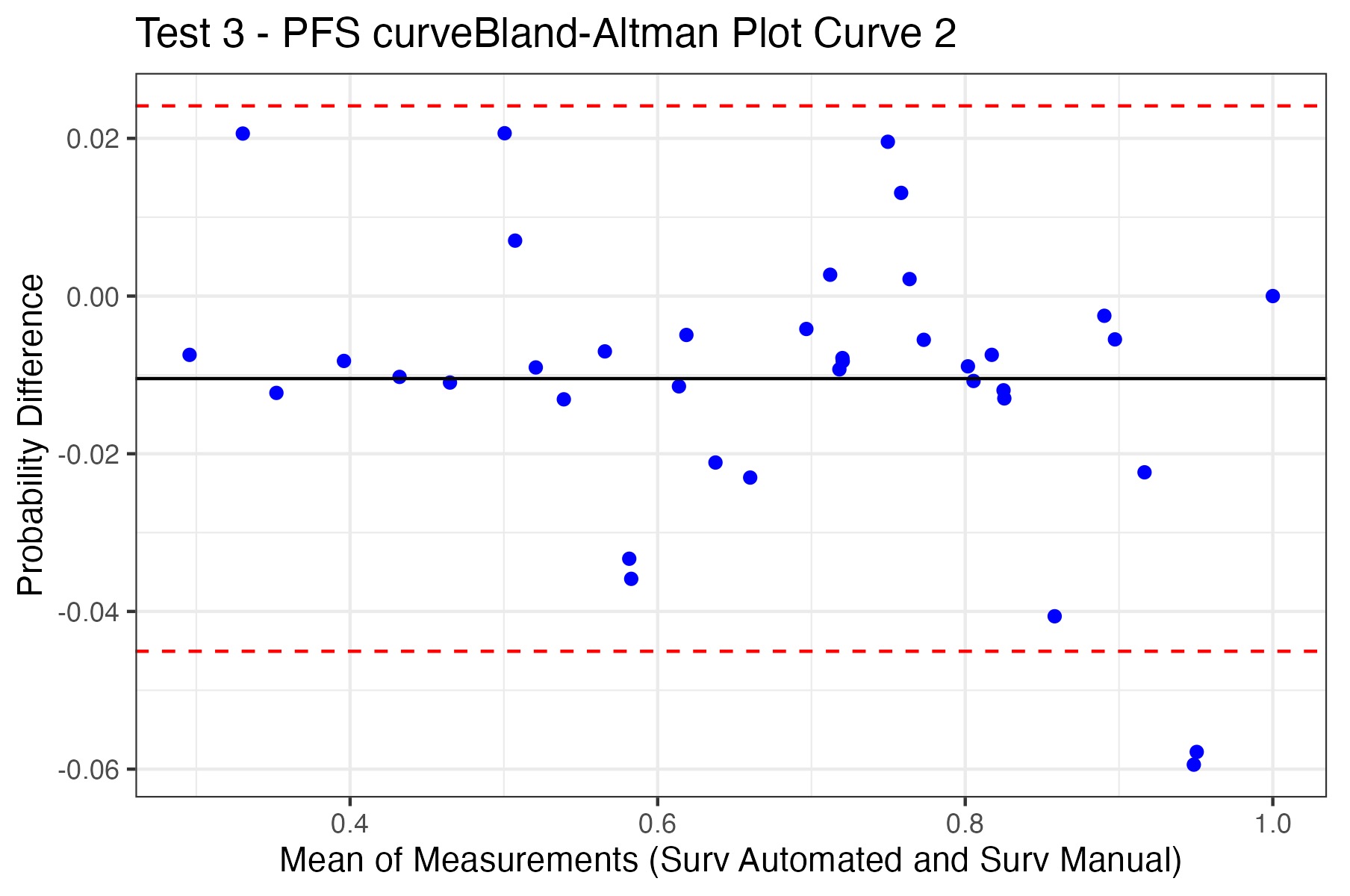

Supplement: Supplementary file 7 — Supplementary Material 7: Fig. S7 Bland Alman plots illustrating the agreement between manual and automated digitization Test 3 PFS curve 1 and curve 2. [file 12874_2024_2273_MOESM7_ESM.zip › Figure S7B.jpg]

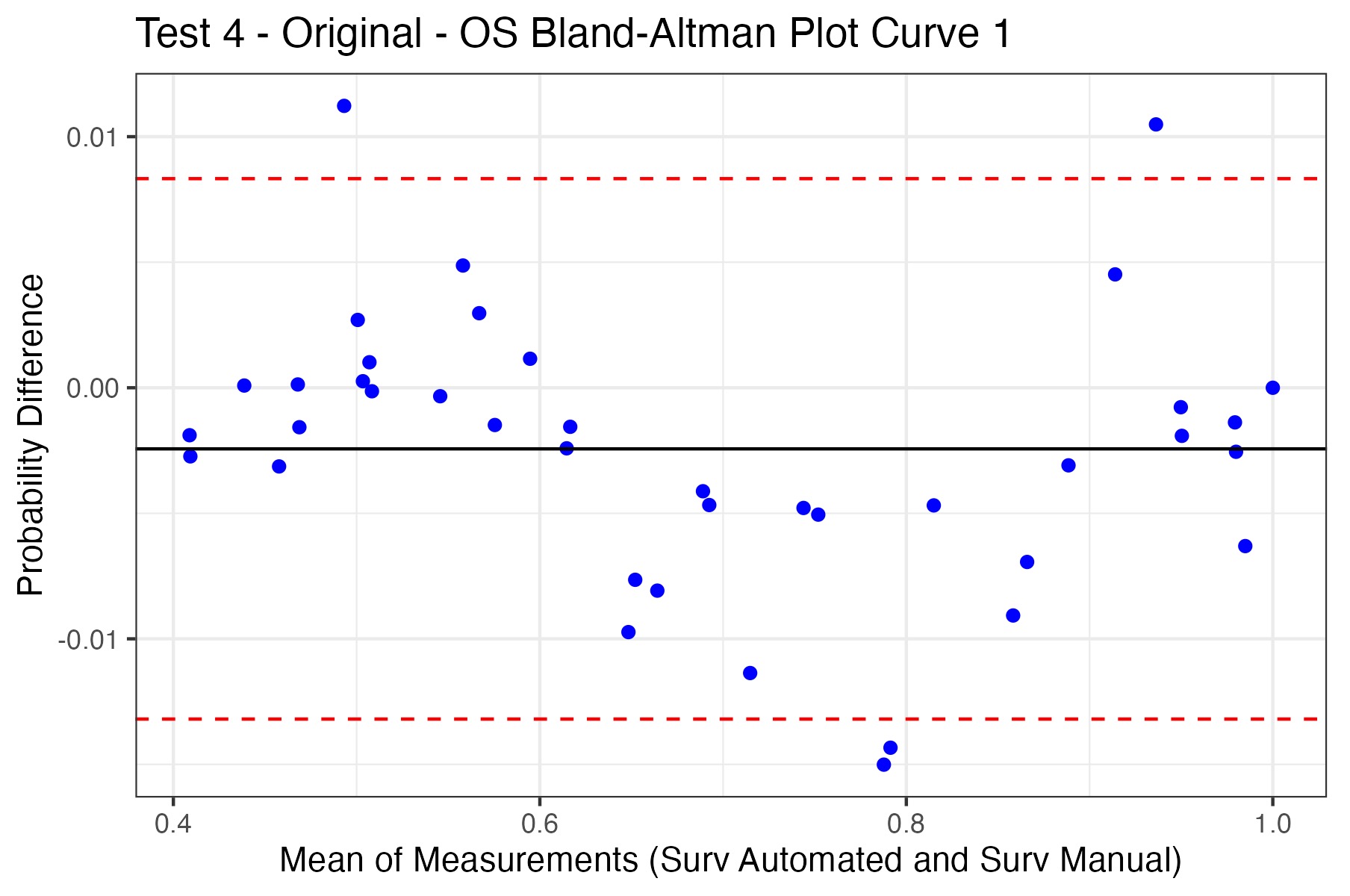

Supplement: Supplementary file 8 — Supplementary Material 8: Fig. S8 Bland Alman plots illustrating the agreement between manual and automated digitization Test 4 OS curve 1 and curve 2. [file 12874_2024_2273_MOESM8_ESM.zip › Figure S8A.jpg]

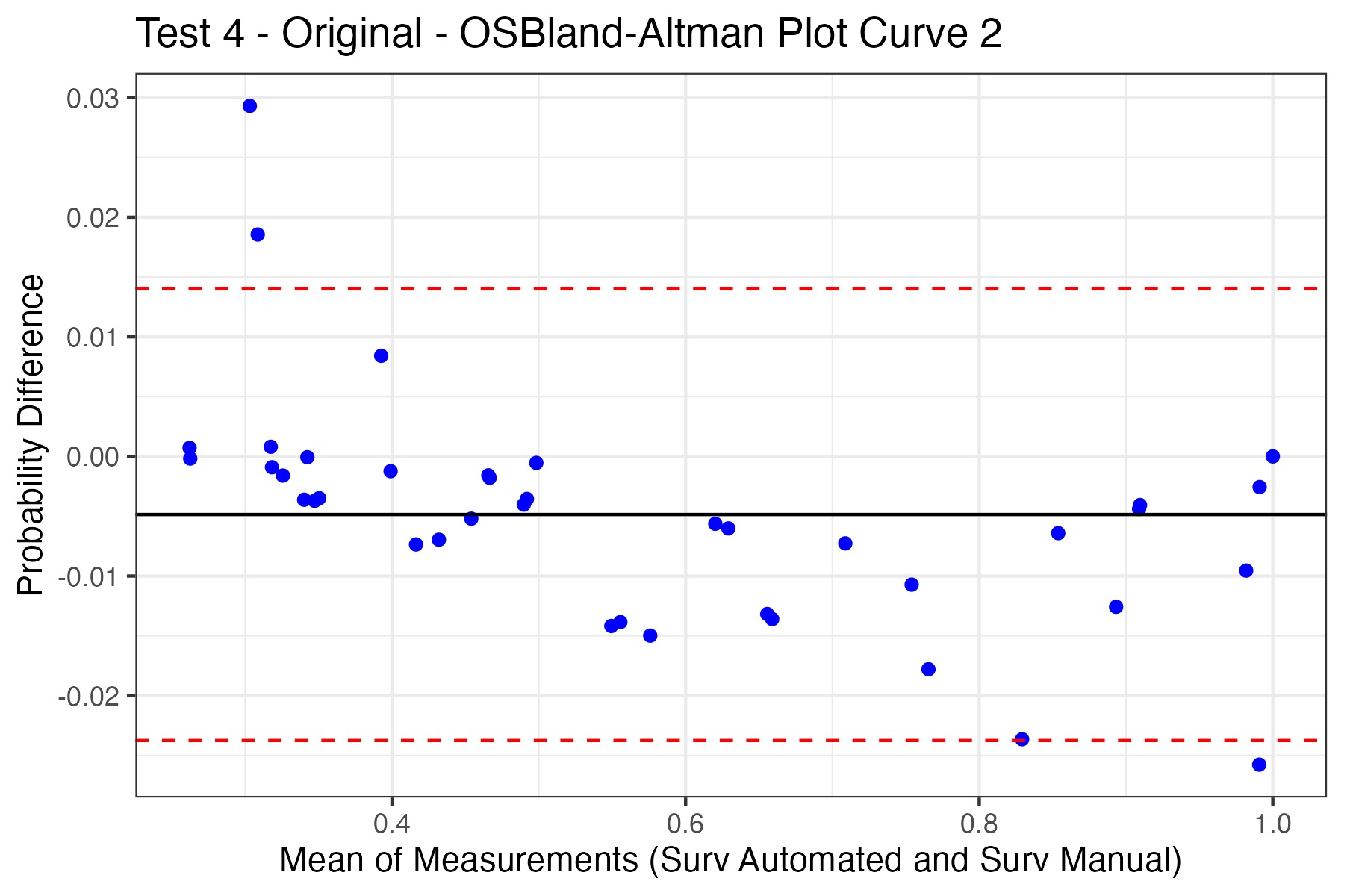

Supplement: Supplementary file 8 — Supplementary Material 8: Fig. S8 Bland Alman plots illustrating the agreement between manual and automated digitization Test 4 OS curve 1 and curve 2. [file 12874_2024_2273_MOESM8_ESM.zip › Figure S8B.jpg]

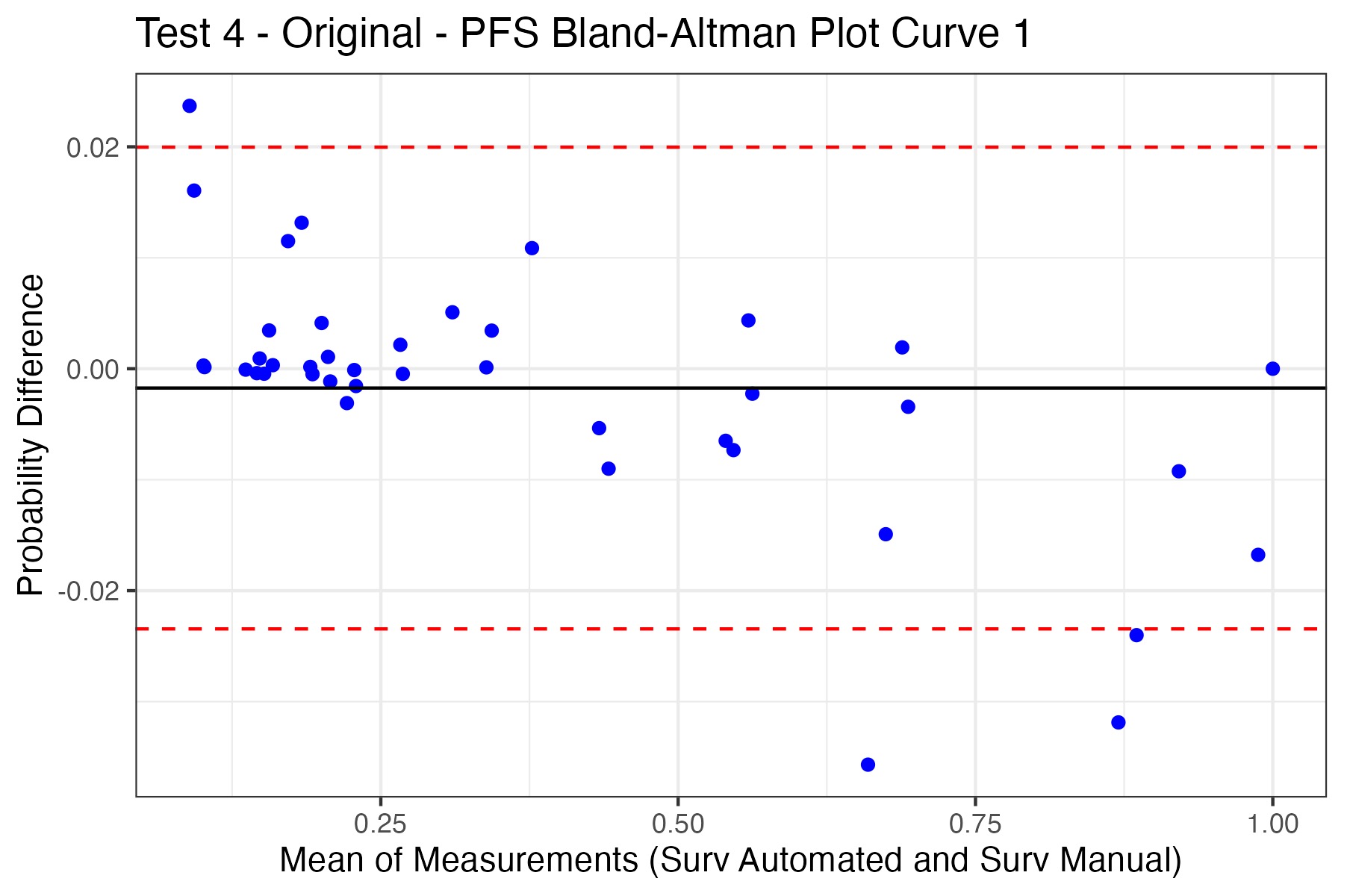

Supplement: Supplementary file 9 — Supplementary Material 9: Fig. S9 Bland Alman plots illustrating the agreement between manual and automated digitization Test 4 PFS curve 1 and curve 2. [file 12874_2024_2273_MOESM9_ESM.zip › Figure S9A.jpg]

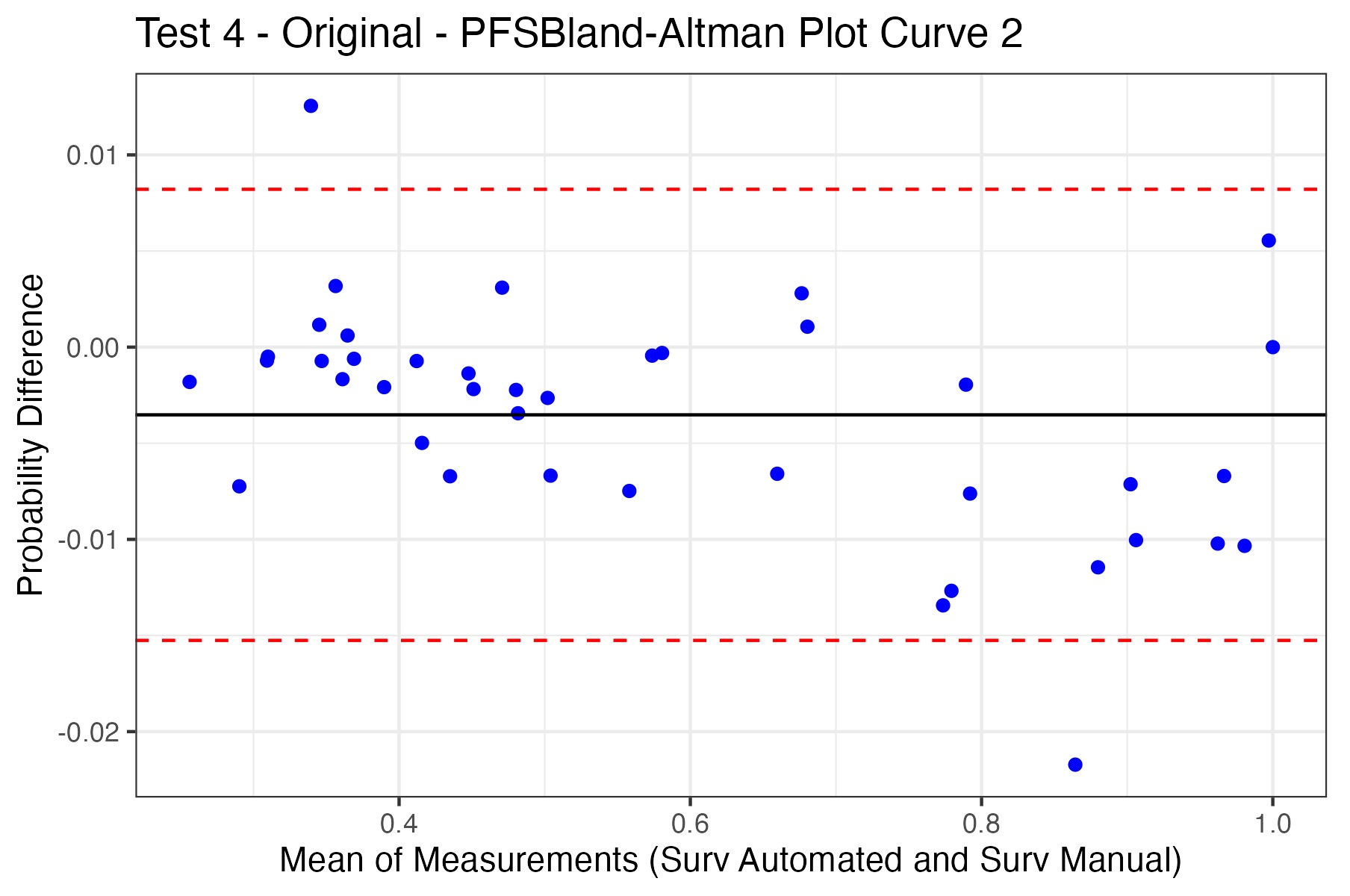

Supplement: Supplementary file 9 — Supplementary Material 9: Fig. S9 Bland Alman plots illustrating the agreement between manual and automated digitization Test 4 PFS curve 1 and curve 2. [file 12874_2024_2273_MOESM9_ESM.zip › Figure S9B.jpg]
